# Supplementary material for: Hemispheric asymmetry of tau pathology is related to asymmetric amyloid deposition in Alzheimer’s Disease
Source: Nat Commun. 2025 Sep 5;16:8232. doi: 10.1038/s41467-025-63564-2 (PMC12413461; doi:10.1038/s41467-025-63564-2)
Supplement: Supplementary file 1 — Supplementary Information [file 41467_2025_63564_MOESM1_ESM.pdf]

Supplementary S1 – Extended methods

Inclusion and exclusion criteria for the participants

The Swedish BioFINDER-2 study enrolls participants in five sub-cohorts. Cohort A and B includes neurologically and cognitively healthy controls. The inclusion criteria are: i) ages 40-65 years (cohort A) and ages 66-100 years (cohort B); ii) absence of cognitive symptoms as assessed by a physician with special interest in cognitive disorders; iii) Mini Mental State Examination (MMSE) score 27-30 (cohort A) or 26-30 (cohort B) at screening visit; iv) do not fulfill the criteria for MCI or any dementia according to DSM-5[1]; v) fluent in Swedish.

Cohort C comprises participants with subjective cognitive deficits (SCD), or mild cognitive impairment (MCI; defined as a performance of <-1.5 SD below reference mean in at least one cognitive domain, see [2] for further details). Inclusion criteria are: i) Age 40-100 years; ii) referred to the memory clinics due to cognitive symptoms; iii) MMSE score of 24 – 30 points; iv) does not fulfill the criteria for any dementia (major neurocognitive disorder) according to DSM-5, v) fluent in Swedish.

Cohort D consists of participants with dementia due to AD. Inclusion criteria are: i) Age 40-100 years; ii) referred to the memory clinics due to cognitive symptoms; iii) MMSE score of > 12 points; iv) fulfill the DSM-5 criteria for dementia (major neurocognitive disorder) due to Alzheimer's disease[1]; v) fluent in Swedish.

Cohort E covers other non-AD dementias and neurodegenerative disorders. Inclusion criteria are: i) Age 40-100 years; ii) fulfillment of criteria for dementia (major neurocognitive disorder) due to Frototemporal dementia (FTD), Parkinson's disease with dementia (PDD), dementia with Lewy bodies (DLB) or subcortical VaD accordingly to the DSM-5 alternatively the criteria for Parkinson's disease (PD),[3] progressive supranuclear palsy (PSP),[4] multiple system

atrophy (MSA),[5] corticobasal syndrome (CBS)[6] or semantic variant primary progressive aphasia (svPPA)[7]; iii) fluent in Swedish. Exclusion criteria for all sub-cohorts are: i) significant unstable systemic illness that makes it difficult to participate in the study; ii) current significant alcohol or substance misuse; iii) refusing lumbar puncture, MRI or PET.

The participants in the present study had been enrolled in either cohort A, B, or C (only people with SCD) of the BioFINDER-2 study for the cognitively unimpaired (CU) group, in cohort C for the MCI group, or D for the AD group. All participants were assessed by physicians with expertise in dementia disorders.

Regions of interest

The meta regions of interest (meta-ROIs) included known regions relevant to neuropathological progression in neurodegenerative diseases, such as Braak staging for tau pathology and Aβ staging based on its progression.[8–10] The meta-ROIs also included commonly used temporal meta-ROI which is based on Braak I-IV and global (i.e., whole-brain) meta-ROI. See Table S1.1 for detailed overview of the meta-ROIs and the regions involved.

Table S1.1. Meta regions of interest.

Meta-ROI	Regions involved
**Global**	*whole brain* (i.e., all Desikan-Killiany regions)
**Temporal**	entorhinal cortex, parahippocampal cortex, fusiform cortex, amygdala, inferior temporal cortex, middle temporal cortex
**Braak I-II**	entorhinal cortex
**Braak III-IV**	parahippocampal cortex, fusiform cortex, amygdala, inferior temporal cortex, middle temporal cortex
**Braak V-VI**	caudal anterior cingulate cortex, caudal middle frontal cortex, cuneus, inferior parietal cortex, isthmus cingulate cortex, lateral occipital cortex, lateral orbitofrontal cortex, lingual cortex, medial orbitofrontal cortex, paracentral cortex, pars opercularis, pars triangularis, pars orbitalis, pericalcarine cortex, postcentral cortex, posterior cingulate cortex, precentral cortex, precuneus, rostral anterior cingulate cortex, rostral middle frontal cortex, superior frontal cortex, superior parietal cortex, superior temporal cortex, supramarginal cortex, frontal pole, temporal pole, transverse temporal cortex, insula

**Early-Aβ**	precuneus, posterior cingulate cortex, isthmus cingulate cortex, insula, medial orbitofrontal cortex, lateral orbitofrontal cortex
**Intermediate-Aβ**	banks of superior temporal sulcus, caudal middle frontal cortex, cuneus, frontal pole, fusiform cortex, inferior parietal cortex, inferior temporal cortex, lateral occipital cortex, middle temporal cortex, parahippocampal cortex, pars opercularis, pars orbitalis, pars triangularis, putamen, rostral anterior cingulate cortex, rostral middle frontal cortex, superior frontal cortex, superior parietal cortex, superior temporal cortex, supramarginal cortex
**Late-Aβ**	lingual cortex, pericalcarine cortex, paracentral cortex, precentral cortex, postcentral cortex
**Neocortical composite**	caudal anterior cingulate cortex, caudal middle frontal cortex, frontal pole, inferior parietal cortex, isthmus cingulate cortex, lateral orbitofrontal cortex, medial orbitofrontal cortex, middle temporal cortex, pars opercularis, pars orbitalis, pars triangularis, posterior cingulate cortex, precuneus, rostral anterior cingulate cortex, rostral middle frontal cortex, superior frontal cortex, superior parietal cortex, superior temporal cortex, supramarginal cortex

Structural connectivity, microstructural integrity, and functional connectivity

The estimation of structural connectivity (SC) was performed using NiPype,[11] Mrtrix3,[12] FSL[13] and FreeSurfer[14] software packages. Firstly, a single set of response functions for white matter (WM), gray matter (GM) and cerebrospinal fluid (CSF) were estimated by "dhollander" algorithm[15,16] using pre-processed dMRI data from 60 CU A-T- and 40 CU A+T- participants of the BioFinder2 (BF2) cohort. These were then utilised to estimate fiber orientation distributions (FOD) based on multi-shell multi-tissue Constrained Spherical Deconvolution (CSD)[17] using "msmt_csd" algorithm,[18] which enables to calculate three separate FODs for three tissue types (i.e., WM, GM, CSF) based on multi-shell dMRI data. Second, a five-tissue-type (5TT) segmented tissue image was generated based on Hybrid Surface and Volume Segmentation (HSVS) using "hsvs" algorithm[19] which uses FreeSurfer and FSL to create segmentations of different tissue types (i.e, cortical GM, sub-cortical GM, WM, CSF, and optionally pathological tissue). 5TT image is important for employing anatomical constraints for later fiber tracking i.e. it increases biological plausibility of the tractogram. After that, the

5TT and T1-weighted images are co-registered to the dMRI data using FSL's "flirt"[20,21] with the average of the not gradient weighted b0 dMRI data. Third, Anatomically-Constrained Tractography (ACT) was performed using a probabilistic "iFOD2" tracking algorithm[22,23] by estimating 10 million streamlines with applying the 5TT image and dynamic determination of seed points.[24] Following that, to reduce the bias in overestimation of streamlines compared to biological WM fibres, Spherical-deconvolution Informed Filtering of Tractograms 2 (SIFT2) method[24] was utilised to calculate weights for all streamlines. Structural connectivity (SC) matrix was then generated based on Desikan-Killiany atlas[25] and ASEG protocol[26] (i.e., 84 regions from FreeSurfer's "aparcaseg") using the sum of SIFT2-weighted streamlines as weights of the edges.[27]

For estimating microstructural integrity, diffusion tensor imaging (DTI) was applied to the dMRI data using weighted least-squares method[28] and removing the b2500 volumes from dMRI data beforehand (i.e., using only b0, b100, b1000 shells). Then, maps of fractional anisotropy (FA) and mean diffusivity (MD) were calculated from the tensor model.[29] White matter tract segmentation was performed using TractSeg, a convolutional neural network-based approach that directly segments tracts from fiber orientation distribution function peaks.[30] The algorithm generated bundle segmentations of the main inter-hemispheric white matter tracts (corpus callosum, forceps major, forceps minor), from which mean FA and MD values were extracted for each tract.

Functional connectivity (FC) was estimated using Nilearn software.[31] FC were constructed from the subject-space pre-processed resting state fMRI data by extracting time series data[32] from the same Desikan-Killiany regions as previously done for SC. After that, Pearson correlation with Fisher's z-transformation[33] was applied between all brain regions for calculating FC.

Normative brain connectivity masks

For analyses investigating brain connectivity, normative brain masks were created from 294 age-matched, pathology-free healthy controls from the BioFINDER-2 cohort. These masks were generated by binarizing the averaged functional and structural connectomes to retain only the top 10% strongest connections, ensuring preservation of biologically meaningful pathways while effectively reducing noise from weaker, potentially spurious connections. The 10% threshold was chosen to be sufficiently strict to focus on the most robust connections, yet not so restrictive as to segregate or exclude any brain regions; all regions remain represented in the resulting masks. Whole-brain connectivity masks were first created for functional connectivity (Fig. S1.1a) and structural connectivity (Fig. S1.1b) analyses. Inter-hemispheric connectivity masks were subsequently defined by restricting to only between-hemisphere connections for functional connectivity (Fig. S1.1c) and structural connectivity (Fig. S1.1d).

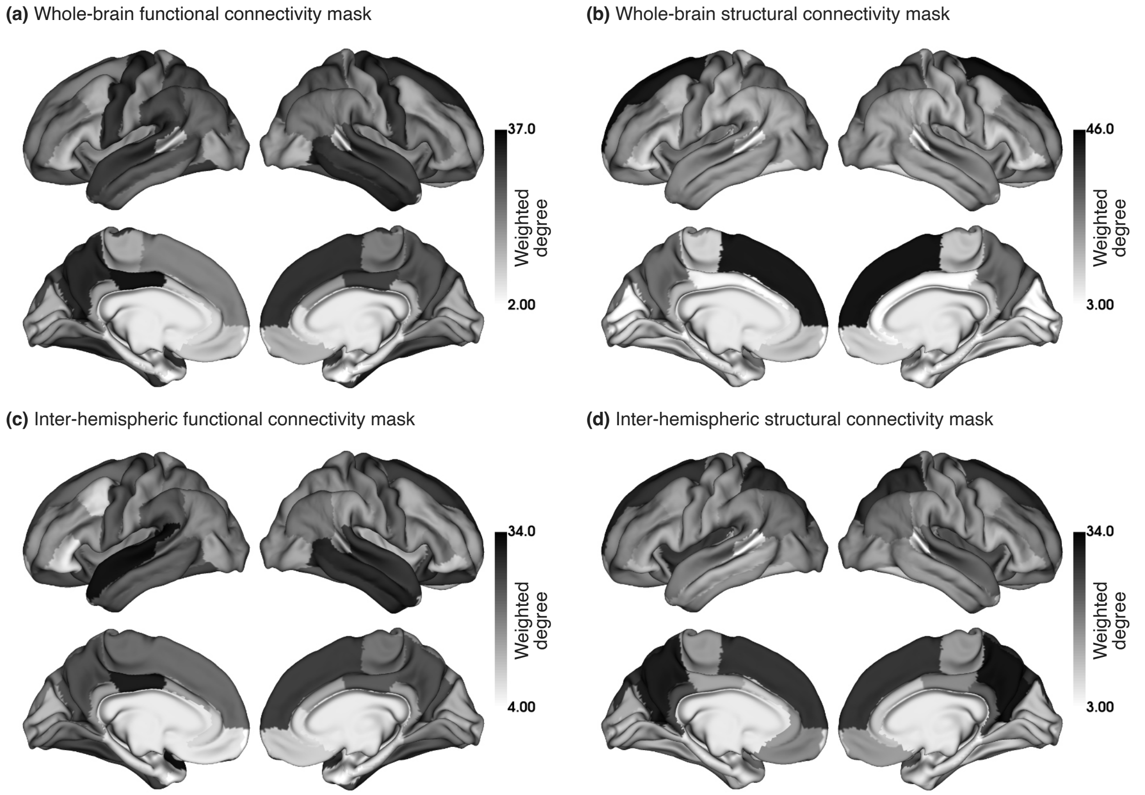

Figure S1.1. Weighted degree distribution of connectivity masks derived from healthy controls. Brain connectivity masks showing weighted degree (number of connections per region) for (a) whole-brain functional connectivity, (b) whole-brain structural connectivity, (c) inter-hemispheric functional connectivity, and (d) inter-

hemispheric structural connectivity.
All masks represent the top 10% strongest connections identified in 294 pathology-free BioFINDER-2 controls.
Color scale indicates weighted degree values.

Supplementary S2 – Extended results

Mean diffusivity across the main tracts connecting the two hemispheres

Similarly to the analysis of comparing fractional anisotropy of the three white matter tracts between the tau asymmetry groups, mean diffusivity revealed no significant differences between the subjects displaying asymmetrical tau distribution compared to individuals with symmetric tau (Fig. S2.1) – in the corpus callosum (S-LA: β=-0.065, 95%CI=[-0.311; 0.181], p_{Bonf}>0.9; S-RA: β=0.040, 95%CI=[-0.329; 0.408], p_{Bonf}>0.9), forceps major (S-LA: β=-0.013, 95%CI=[-0.264; 0.238], p_{Bonf}>0.9; S-RA: β=0.210, 95%CI=[-0.159; 0.578], p_{Bonf}=0.790), or forceps minor (S-LA: β=0.112, 95%CI=[-0.137; 0.361], p_{Bonf}>0.9; S-RA: β=0.148, 95%CI=[-0.223; 0.520], p_{Bonf}>0.9).

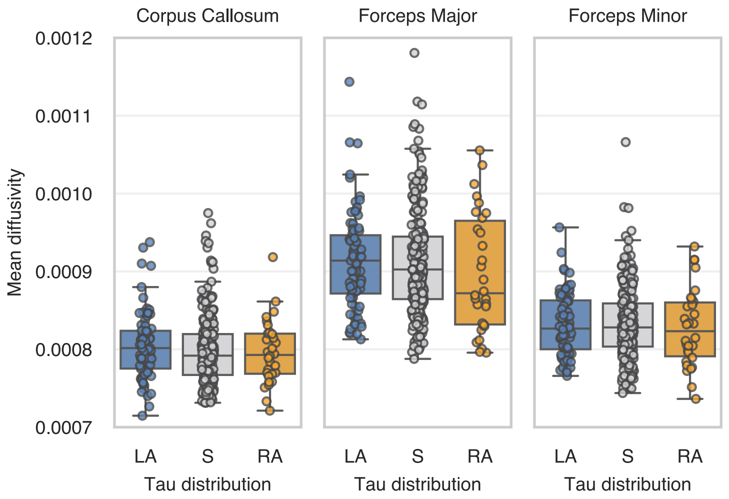

Figure S2.1. Average mean diffusivity across three main inter-hemispheric white matter tracts. Boxplots represent mean diffusivity across the three tau asymmetry groups, where the groups were statistically compared using ordinary least squares multiple linear regression models (mean diffusivity ~ age + sex + global tau load + group), with the significance levels Bonferroni-corrected for the number of group comparisons. The horizontal line within each box indicates the median, while the lower and upper box edges denote the first and third quartiles, respectively. Whiskers extend to 1.5 times the interquartile range, and dots represent individual data points. LA, left tau asymmetric; S, tau symmetric; RA, right tau asymmetric.

Region-specific associations between connectivity and tau pathology

To assess region-specific relationships between inter-hemispheric brain connectivity and tau pathology, linear regressions were performed within the A+T+ group, examining associations of homotopic connectivity with both bilateral tau load and absolute tau laterality. After adjusting for age and sex, higher tau burden was significantly associated with lower functional connectivity in occipital and temporal regions (Fig. S2.2), with the strongest effects observed in the superior parietal (β=-0.299, p_{FDR}<0.001), fusiform (β=-0.287, p_{FDR}<0.001), and lateral occipital (β=-0.230, p_{FDR}<0.001) gyri. For structural connectivity, lower connectivity was significantly related to higher tau burden in regions across the neocortex, particularly in the superior frontal (β=-0.229, p_{FDR}<0.001), superior parietal (β=-0.218, p_{FDR}<0.001), and precuneus (β=-0.213, p_{FDR}<0.001) gyri. Temporal regions did not show significant associations, except for a positive relationship in the superior temporal gyrus (β=0.182, p_{FDR}=0.002). In contrast, when examining associations between homotopic connectivity and absolute tau laterality (adjusted for age, sex, and bilateral tau load), no regions showed statistically significant relationships in functional connectivity (all p_{FDR}>0.6) or structural connectivity (all p_{FDR}>0.6).

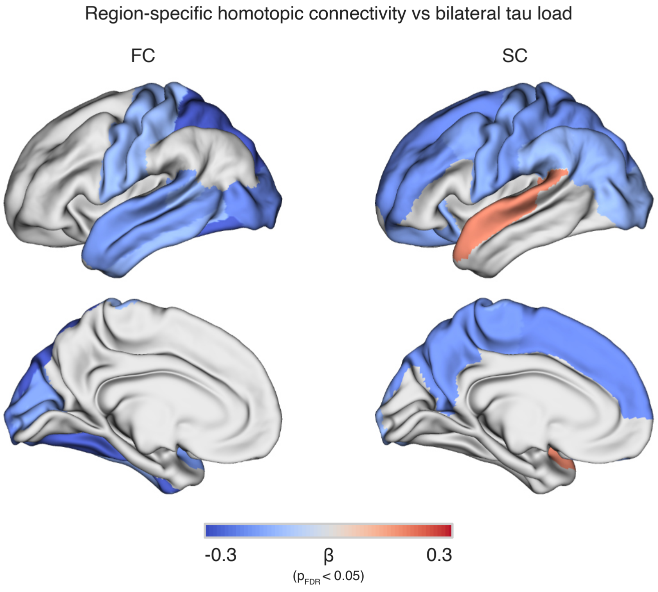

Figure S2.2. Region-specific associations between homotopic connectivity and bilateral tau load. Ordinary least squares multiple linear regressions were employed to estimate the effect of regional connectivity on tau load with the models adjusted for age and sex and FDR-corrected. FC, functional connectivity; SC, structural connectivity; FDR, false discovery rate.

Network Based Statistics

Whole-brain connectome analysis using Network Based Statistics (NBS) showed some minor differences between the tau asymmetry groups (Table S2.1; Fig. S2.3a). Higher functional connectivity in the left asymmetric group compared to the symmetric group was found in a cluster of nodes primarily located in the right hemisphere which also included a few inter-hemispheric connections (threshold=3.0; C=9, p=0.018). The right asymmetric group showed reduced structural connectivity compared to the symmetric group in a cluster of nodes within the right hemisphere (threshold=3.0; C=4, p=0.016). However, the results were not consistent across statistical thresholds. Similar results were also found when the normative connectivity masks were not used (i.e., all connections were included in the analyses). Structural connectivity displayed more consistent difference between the right asymmetric and symmetric groups across the different statistical thresholds but, again, almost exclusively within the right hemisphere. This suggest that the lower structural connectivity in the right asymmetric group is an effect of the higher tau burden in that hemisphere (Fig. S2.3b). Therefore, we considered

these results not entirely reliable. Moreover, these differences are likely due to the unbalanced tau burden in the affected hemisphere for subjects with asymmetric tau distribution which we were not able properly adjust for at the single node level in NBS (i.e., we adjusted for global average tau uptake).

Table S2.1. One-sided NBS tests of functional and structural connectivity between tau asymmetry groups. Each column represents a contrast between groups and each row shows the t-statistic threshold (t) used. Each cell represents the maximum detected component size C and the significance level i.e., C (p-value). A, tau asymmetric; LA, left tau asymmetric; S, tau symmetric; RA, right tau asymmetric; FC, functional connectivity; SC, structural connectivity.

**Connectomes masked for top 10% whole-brain connections**						
**FC**	**A > S**	**A < S**	**LA > S**	**LA < S**	**RA > S**	**RA < S**
**t = 2.5**	**19 (p=0.045)**	-	**23 (p=0.041)**	-	11 (p=0.072)	2 (p=0.239)
**t = 3.0**	2 (p=0.077)	-	**9 (p=0.018)**	-	3 (p=0.056)	1 (p=0.161)
**t = 3.5**	-	-	1 (p=0.053)	-	1 (p=0.054)	-
**SC**	**A > S**	**A < S**	**LA > S**	**LA < S**	**RA > S**	**RA < S**
**t = 2.5**	1 (p=0.684)	5 (p=0.104)	3 (p=0.231)	6 (p=0.074)	2 (p=0.423)	**13 (p=0.031)**
**t = 3.0**	-	2 (p=0.081)	1 (p=0.384)	1 (p=0.241)	2 (p=0.156)	**4 (p=0.016)**
**t = 3.5**	-	1 (p=0.073)	1 (p=0.127)	-	-	-
**Sensitivity analysis: connectomes unmasked**						
**FC**	**A > S**	**A < S**	**LA > S**	**LA < S**	**RA > S**	**RA < S**
**t = 2.5**	72 (p=0.077)	-	**94 (p=0.050)**	3 (p=0.489)	34 (p=0.158)	10 (p=0.295)
**t = 3.0**	11 (p=0.082)	-	**26 (p=0.038)**	-	12 (p=0.079)	1 (p=0.404)
**t = 3.5**	1 (p=0.155)	-	2 (p=0.076)	-	1 (p=0.167)	-
**SC**	**A > S**	**A < S**	**LA > S**	**LA < S**	**RA > S**	**RA < S**
**t = 2.5**	9 (p=0.452)	51 (p=0.062)	14 (p=0.426)	**59 (p=0.038)**	46 (p=0.255)	**50 (p=0.038)**
**t = 3.0**	1 (p=0.780)	7 (p=0.058)	1 (p=0.849)	5 (p=0.064)	7 (p=0.320)	**15 (p=0.012)**
**t = 3.5**	1 (p=0.360)	1 (p=0.165)	1 (p=0.457)	1 (p=0.119)	2 (p=0.364)	**6 (p=0.002)**

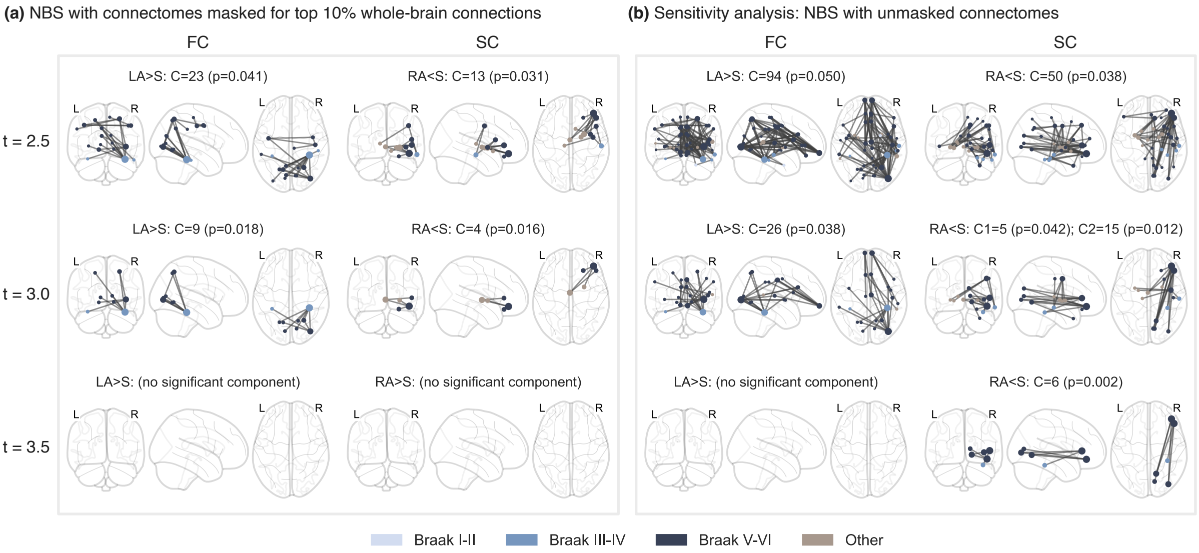

Figure S2.3. Components detected using one-sided NBS analyses between the tau asymmetry groups: (a) With connectomes masked to include only the top 10% whole-brain connections of the age-matched, pathology-free healthy controls (see Supplementary S1 for more information); (b) Without masking the connectomes (i.e., including all possible connections).
FC, functional connectivity; SC, structural connectivity; LA, left tau asymmetric; S, tau symmetric; RA, right tau asymmetric; C, component size; NBS, Network Based Statistic; t, threshold used in NBS

Average connectivity differences between individuals with elevated tau burden and healthy controls

To evaluate general connectivity disruptions associated with elevated tau pathology, we compared functional and structural connectivity between the 452 A+T+ individuals and 272 A-T- cognitively unimpaired controls. Connectivity matrices were masked using normative whole-brain and inter-hemispheric masks (see Supplementary S1). Total whole-brain connectivity (averaged across all connections) was significantly reduced in A+T+ individuals (Fig. S2.4a) for both functional connectivity (n=612, β=-0.184, 95%CI=[-0.349; -0.019], p=0.028) and structural connectivity (n=646, β=-0.387, 95%CI=[-0.531; -0.243], p<0.001). Average inter-hemispheric connectivity was similarly diminished in the A+T+ group (Fig. S2.4b) for functional connectivity (β=-0.186, 95%CI=[-0.351; -0.021], p=0.027) and structural connectivity (β=-0.190, 95%CI=[-0.338; -0.042], p=0.012). All models were adjusted for age

and sex. These results demonstrate that tau pathology is associated with broad reduction in brain connectivity, providing critical context for investigating tau distribution related effects.

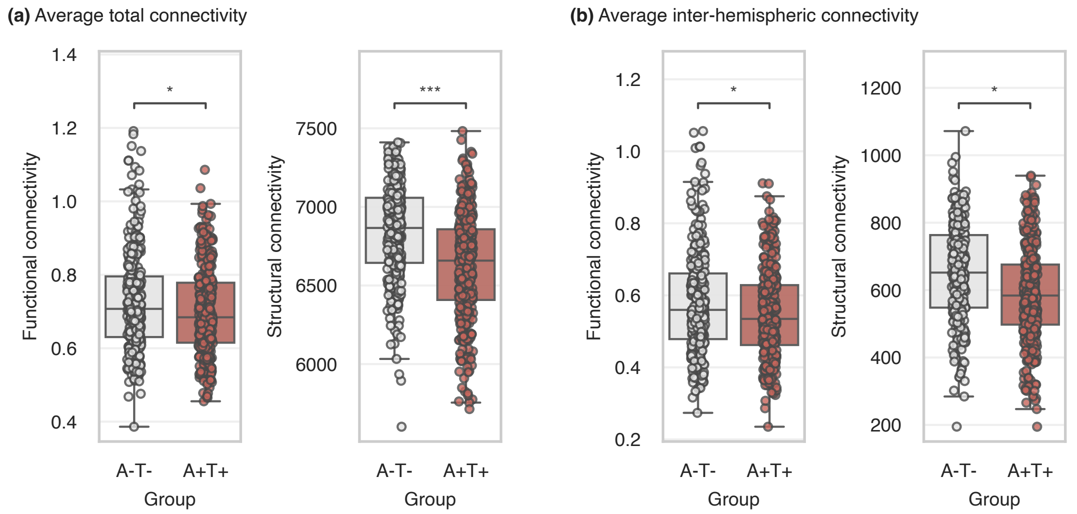

Figure S2.4. Average functional and structural connectivity between the cross-sectional A+T+ sample and A-T- healthy controls: (a) Across the whole brain; (b) Within the inter-hemispheric connections. The connectomes were masked with the corresponding normative masks. Both panels show a statistical comparison of brain connectivity with ordinary least squares multiple linear regressions where the group is binarized predictor and models are adjusted for age and sex. A-T-, healthy pathology-free controls; A+T+, individuals with evidence of amyloid-beta and tau pathology; *, $p<0.05$; ***, $p<0.001$.

Association between tau laterality and brain connectivity within meta-ROIs defined by tau burden severity

To test whether associations between tau laterality and average brain connectivity were affected by different levels of tau load in different regions, we conducted an additional analysis within three new composite ROIs. These ROIs were defined based on group-average bilateral tau load of the 452 A+T+ subjects. Regions were assigned to the low tau burden ROI if their group-average tau load was below the 25th percentile threshold (i.e., SUVR < 1.259), and to the high tau burden ROI if their tau load exceeded the 75th percentile threshold (i.e., SUVR > 1.637). Regions with values between these thresholds were assigned to the medium tau burden ROI. Next, we investigated average inter-hemispheric (Fig. S2.5) and intra-hemispheric (Fig. S2.6) functional and structural connectivity and their associations with the absolute tau laterality index using linear regression within each of these composite ROIs. All models were

adjusted for age, sex, and bilateral tau load, and p-values were Bonferroni-corrected for the number of composite ROIs (i.e., 3). Inter-hemispheric functional connectivity did not show any significant association with absolute tau laterality in any of the ROIs (Low tau: β=0.006, 95%CI=[-0.132; 0.144], p_{Bonf}>0.900; Medium tau: β=0.068, 95%CI=[-0.058; 0.195], p_{Bonf}=0.860; High tau: β=0.057, 95%CI=[-0.057; 0.170], p_{Bonf}>0.900); neither did structural connectivity (Low tau: β=-0.110, 95%CI=[-0.228; 0.009], p_{Bonf}=0.212; Medium tau: β=-0.033, 95%CI=[-0.144; 0.078], p_{Bonf}>0.900; High tau: β=-0.003, 95%CI=[-0.111; 0.105], p_{Bonf}>0.900). Similarly, there were no significant associations between absolute tau laterality and intra-hemispheric functional connectivity (Low tau: β=0.010, 95%CI=[-0.128; 0.148], p_{Bonf}>0.900; Medium tau: β=0.081, 95%CI=[-0.045; 0.207], p_{Bonf}=0.618; High tau: β=0.082, 95%CI=[-0.031; 0.195], p_{Bonf}=0.468) or structural connectivity (Low tau: β=0.086, 95%CI=[-0.037; 0.209], p_{Bonf}=0.512; Medium tau: β=-0.017, 95%CI=[-0.137; 0.104], p_{Bonf}>0.900; High tau: β=-0.016, 95%CI=[-0.115; 0.084], p_{Bonf}>0.900).

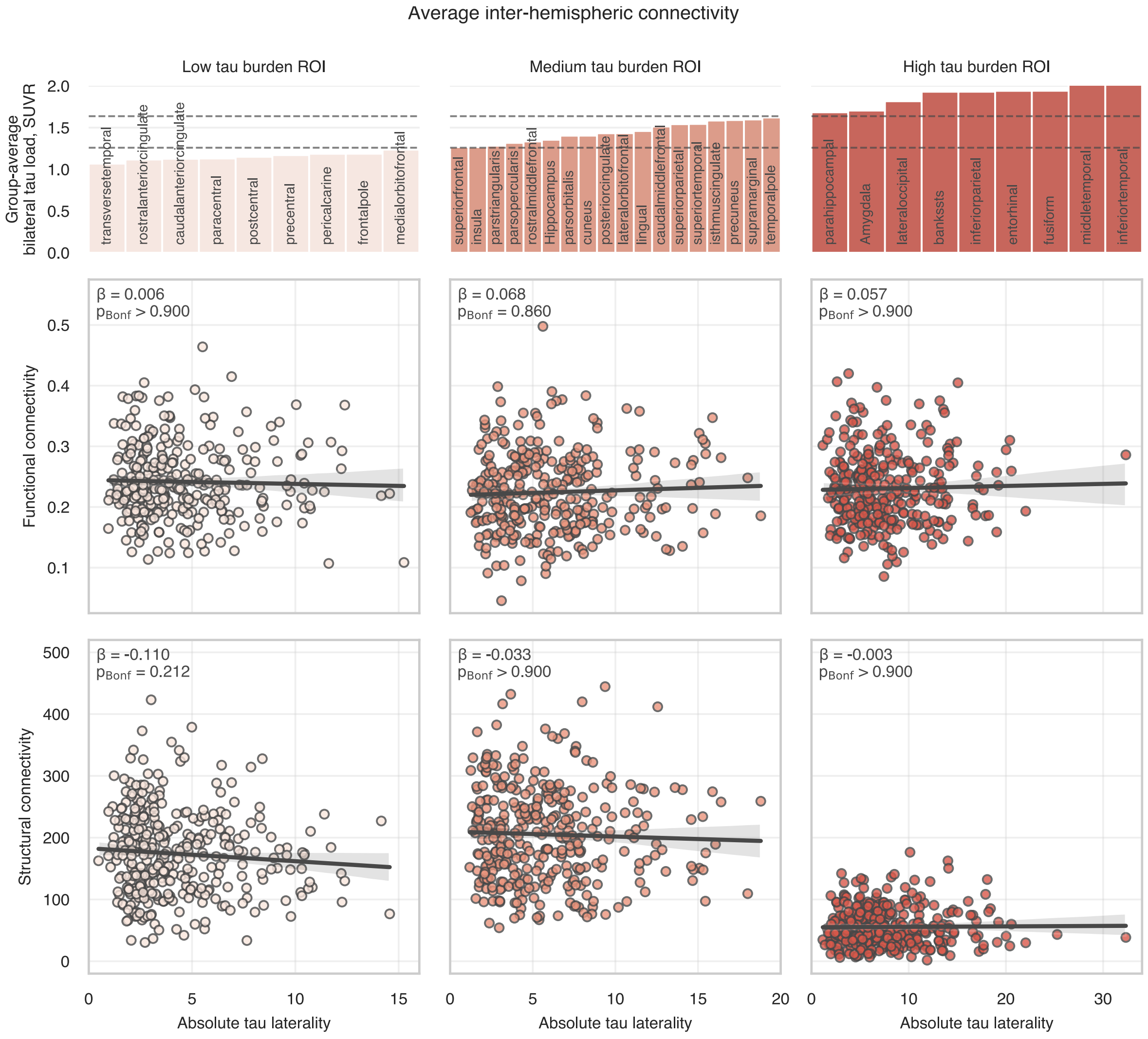

Figure S2.5. Association between absolute tau laterality index and inter-hemispheric functional/structural connectivity across three composite ROIs defined by group-average tau burden severity. Columns represent the three composite ROIs: low tau burden (SUVR < 1.259, 25th percentile), medium tau burden (1.259 ≤ SUVR ≤ 1.637), and high tau burden (SUVR > 1.637, 75th percentile). Regions included in each composite ROI, with group-average tau and thresholds (dashed lines) for low/high tau classification. Middle row displays the analyses with functional connectivity and bottom row with structural connectivity.
All scatterplots show regression lines with 95% confidence intervals, with statistical annotations indicating the standardized effect size and significance level of tau laterality as a predictor of connectivity in ordinary least squares multiple linear regression models (composite-ROI inter-hemispheric connectivity ~ age + sex + composite-ROI bilateral tau load + composite-ROI absolute tau laterality). P-values were Bonferroni-corrected (×3) and annotated. SUVR, standardised uptake value ratio.

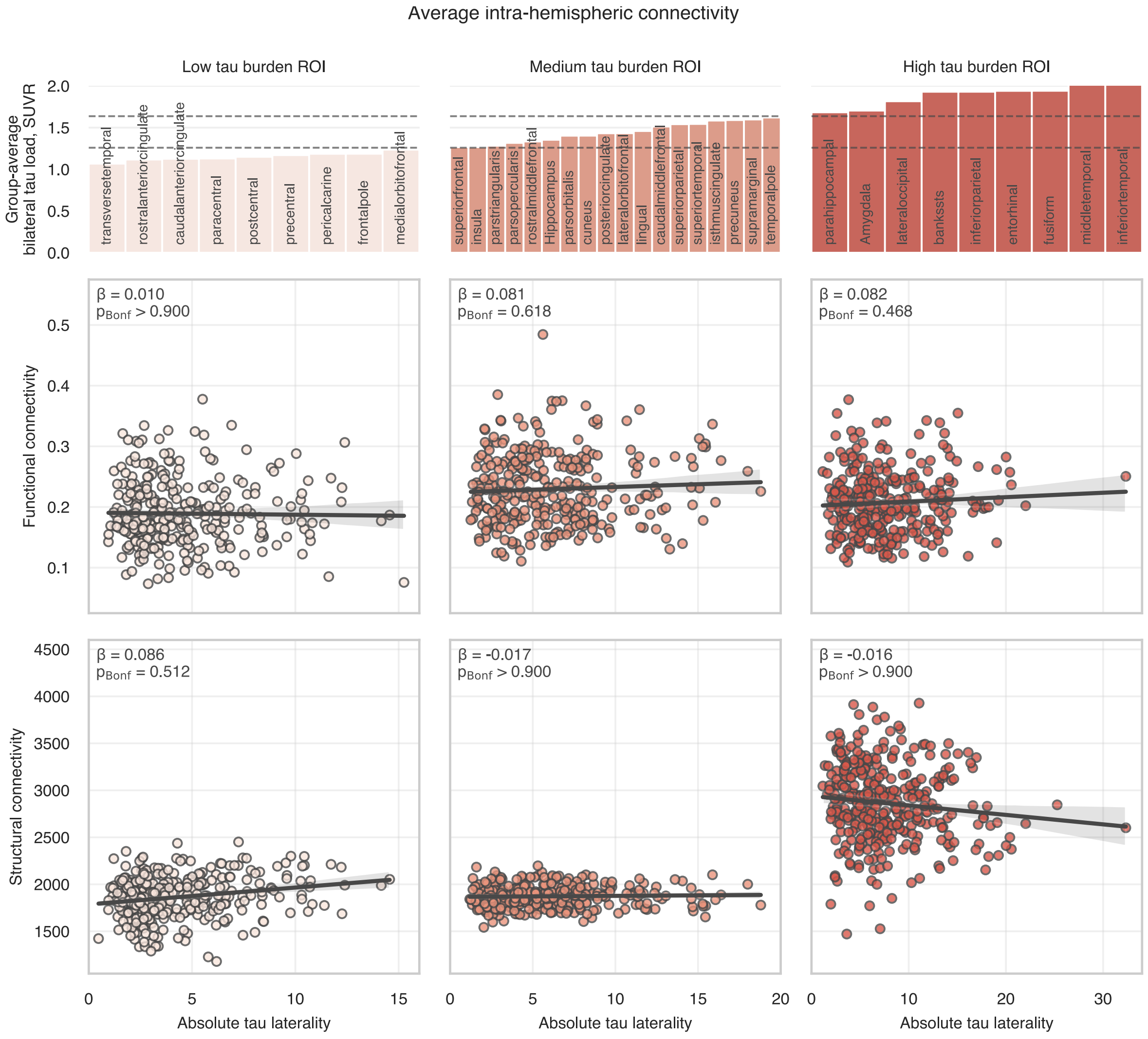

Figure S2.6. Association between absolute tau laterality index and intra-hemispheric functional/structural connectivity across three composite ROIs defined by group-average tau burden severity. Columns represent the three composite ROIs: low tau burden (SUVR < 1.259, 25th percentile), medium tau burden (1.259 ≤ SUVR ≤ 1.637), and high tau burden (SUVR > 1.637, 75th percentile). Regions included in each composite ROI, with group-average tau and thresholds (dashed lines) for low/high tau classification. Middle row displays the analyses with functional connectivity and bottom row with structural connectivity.
All scatterplots show regression lines with 95% confidence intervals, with statistical annotations indicating the standardized effect size and significance level of tau laterality as a predictor of connectivity in ordinary least squares multiple linear regression models (composite-ROI intra-hemispheric connectivity ~ age + sex + composite-ROI bilateral tau load + composite-ROI absolute tau laterality). P-values were Bonferroni-corrected (×3) and annotated. SUVR, standardised uptake value ratio.

Associations between the laterality of Aβ and tau across different regions and meta-ROIs

Between tau laterality and Aβ laterality, an additional set of analyses was performed using different meta-ROIs than used in the main analysis. First, the relationship between global tau

laterality and Aβ laterality at Aβ staging meta-ROIs was assessed (Fig. S2.7a), which resulted the strongest effect size at Intermediate-Aβ meta-ROI (β=0.634, 95%CI=[0.533; 0.735], $p_{Bonf}<0.001$) followed by Late-Aβ (β=0.609, 95%CI=[0.505; 0.713], $p_{Bonf}<0.001$) and Early-Aβ (β=0.547, 95%CI=[0.438; 0.657], $p_{Bonf}<0.001$). Second, the association between global Aβ laterality and tau laterality at different Braak stages was investigated (Fig. S2.7b), with the strongest effect found at Braak III-IV meta-ROI (β=0.655, 95%CI=[0.556; 0.754], $p_{Bonf}<0.001$) followed by Braak V-VI (β=0.594, 95%CI=[0.488; 0.699], $p_{Bonf}<0.001$) and Braak I-II (β=0.443, 95%CI=[0.327; 0.559], $p_{Bonf}<0.001$).

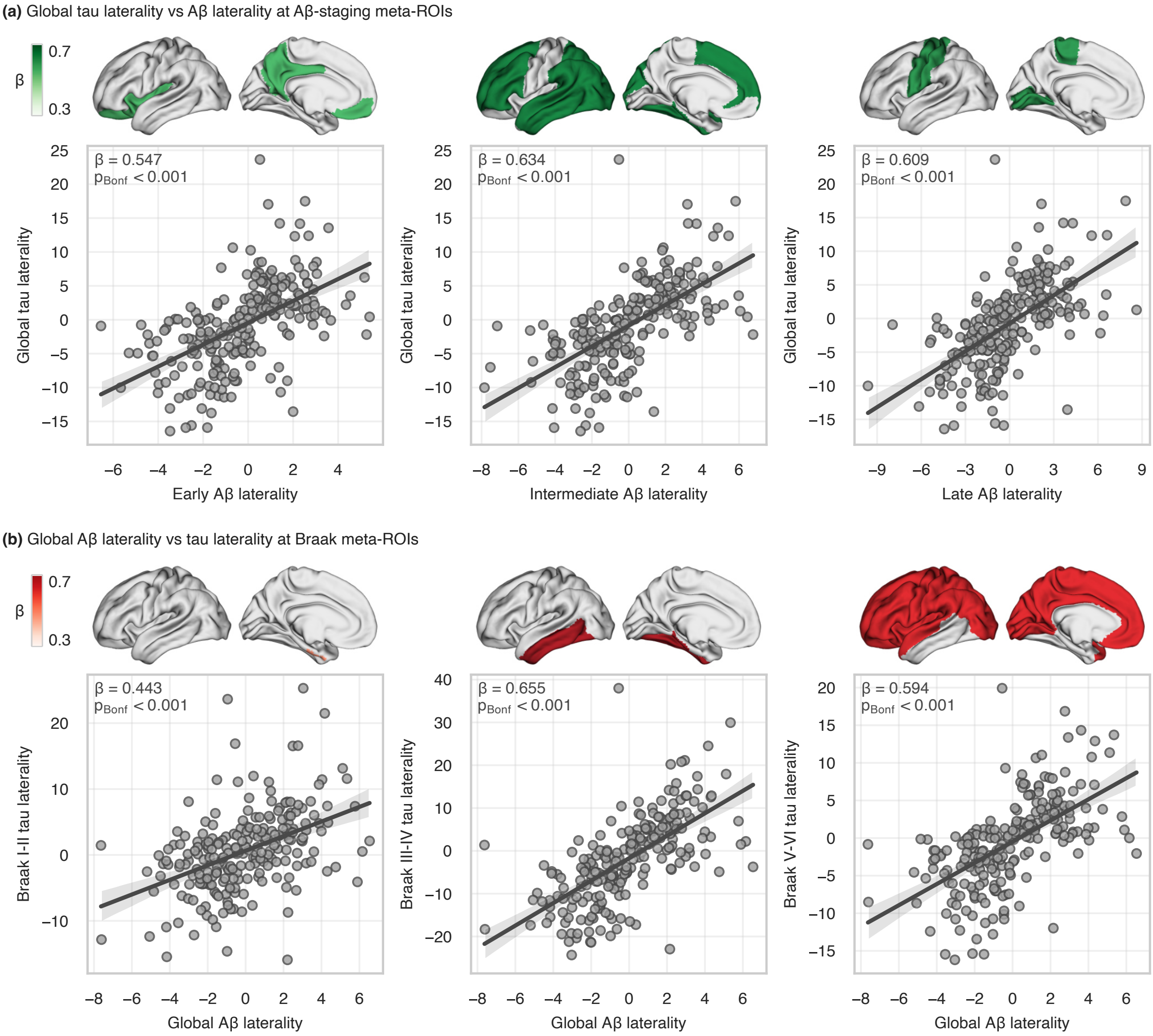

Figure S2.7. Associations between Aβ laterality and tau laterality across meta-ROIs: (a) Global tau laterality vs Aβ laterality at Aβ stages; (b) Global Aβ laterality vs tau laterality at Braak stages.
Both panels show regression lines with 95% confidence intervals, with statistical annotations indicating the

standardized effect size and significance of Aβ laterality as a predictor of tau laterality in an ordinary least squares multiple linear regression model (tau laterality ~ age + sex + Aβ laterality). Bonferroni-corrected p-values (×3) are annotated. Aβ, amyloid-beta.

To examine the regional specificity of Aβ-tau asymmetry relationships, we performed a comprehensive pairwise region-by-region analysis between Aβ laterality and tau laterality (Fig. S2.8). This analysis addressed whether associations between Aβ and tau distributions are regionally specific (i.e., stronger within the same region) or reflect broader cross-regional relationships. Notably, the strongest same-region associations occurred in the temporal lobe, particularly the inferior temporal (β=0.633, p_{FDR}<0.001), fusiform (β=0.613, p_{FDR}<0.001), and middle temporal (β=0.571, p_{FDR}<0.001) gyri. Moreover, tau laterality in temporal regions showed the strongest associations with Aβ laterality across the entire brain. Specifically, tau laterality at inferior temporal gyrus demonstrated the highest average effect size with Aβ laterality across all regions (β_{avg}=0.428), followed by fusiform (β_{avg}=0.405), and middle temporal (β_{avg}=0.405) gyri.

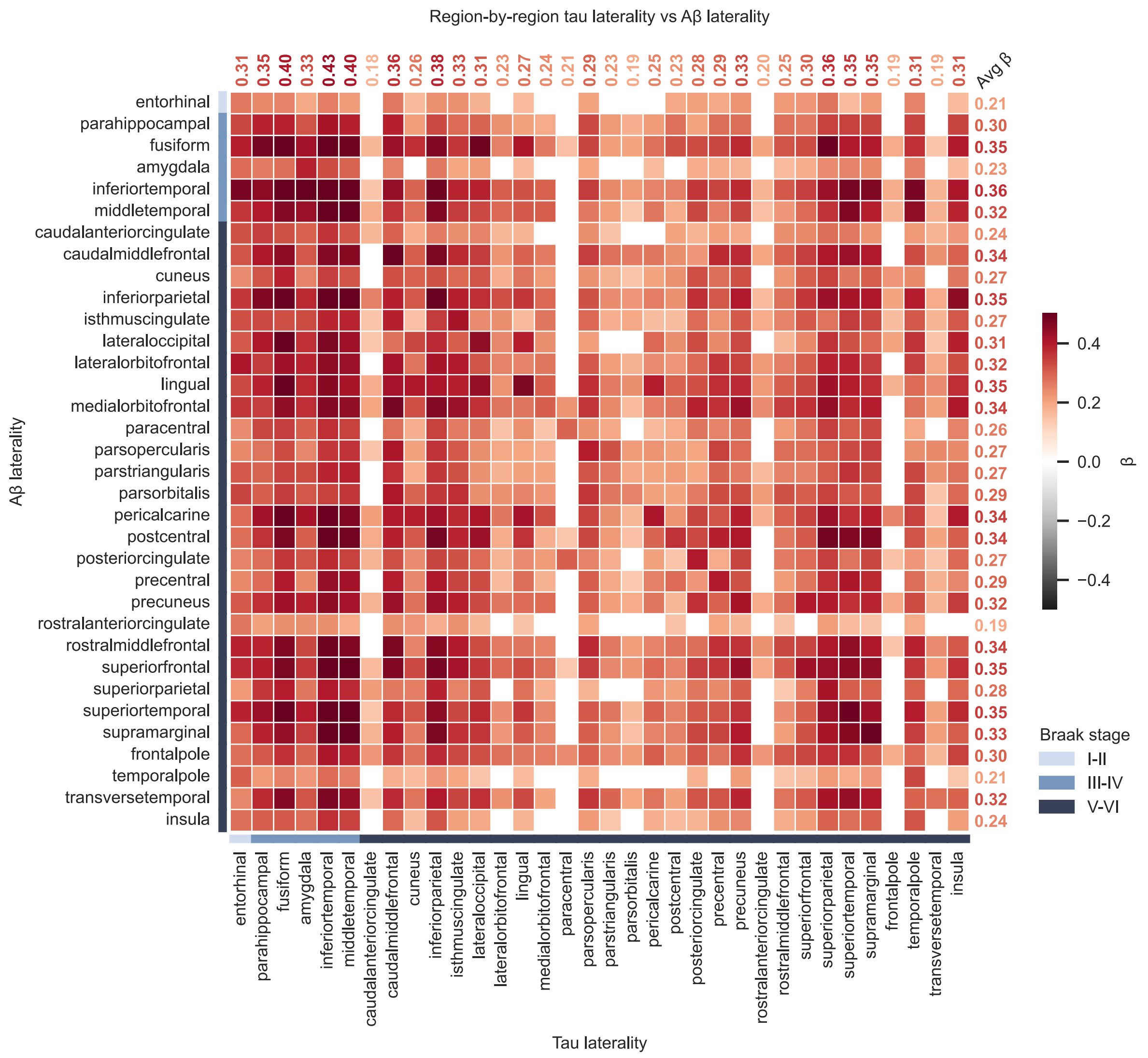

Figure S2.8. Region-by-region associations between Aβ laterality and tau laterality across the brain. All pairs of regions were tested with ordinary least squares multiple linear regression models (tau laterality ~ age + sex + Aβ laterality) and all the coloured values indicate the effect sizes between regions that were statistically significant after FDR-correction. Average effect sizes for all rows and columns are displayed at the edges of the matrix. Aβ, amyloid-beta.

Summary of the longitudinal sample

Table S2.2. Demographics of the longitudinal dataset.
Categorical variables have been presented as 'count (%)', normally distributed continuous variables as 'mean (SD)' and non-normally distributed variables as ‘median [interquartile range]’. All variables were compared between the tau asymmetry groups using either one-way ANOVA, Kurskal-Wallis, or Chi-squared test depending on the type and distribution of the data. T-, tau negative; T+, tau positive; M, male; F, female; CU, cognitively unimpaired; MCI, mild cognitive impairment; AD, Alzheimer’s disease; SUVR, standardized uptake value ratio; LI, laterality index; Aβ, amyloid-beta; MMSE, Mini-Mental State Examination; mPACC, modified Preclinical Alzheimer Cognitive Composite.

	**Longitudinal A+ (n=289)**		
	**T- (n=180)**	**T+ (n=109)**	**P-value**

**Age, years**		73.09 (7.89)	72.08 (7.06)	0.264
**Sex**	**M**	92 (51%)	57 (52%)	0.941
	**F**	88 (49%)	52 (48%)	
**Education, years**		12.78 (4.08)	12.98 (3.68)	0.670
**Diagnosis**	**CU**	116 (64%)	36 (33%)	<0.001
	**MCI**	64 (36%)	69 (63%)	
	**AD**	0 (0%)	4 (4%)	
**Temporal tau, SUVR**		1.19 [1.14,1.24]	1.61 [1.42,2.00]	<0.001
**Absolute Temporal tau LI**		1.31 (1.17)	8.32 (6.10)	<0.001
**Global Aβ, SUVR**		1.27 (0.19)	1.50 (0.20)	<0.001
**ApoE4**	**0**	62 (34%)	17 (16%)	0.001
	**1**	99 (55%)	71 (65%)	
	**2**	19 (11%)	21 (19%)	
**MMSE**		29.00 [27.00,29.25]	27.00 [26.00,29.00]	<0.001
**mPACC**		-0.56 [-1.53,-0.00]	-1.50 [-2.40,-0.63]	<0.001

Longitudinal association between baseline Aβ laterality and changes over time in tau laterality

Table S2.3. Summary of the linear mixed effects model within A+ subsample predicting changes over time in tau laterality with baseline Aβ laterality using the Global meta-ROI.
The statistical significance of the baseline Aβ laterality and its interaction with time are annotated with **green** if it reached $p<0.05$ and **red** if it did not.

A+ (n=289 with a total of 707 datapoints)<br>LME: Tau LI ~ time * (Age_{BAS} + Sex + Aβ LI_{BAS}) + [time | participant]				
**ROI**	**Global**			
	**β**	**SE**	**95% CI**	**p**
**Intercept**	-0.052	0.056	[-0.162, 0.058]	0.350
**Time**	-0.001	0.016	[-0.032, 0.031]	0.964
**Age_{BAS}**	-0.007	0.040	[-0.086, 0.072]	0.864
**Sex**	0.023	0.081	[-0.135, 0.181]	0.776
**Aβ LI_{BAS}**	0.379	0.040	[0.301, 0.458]	**<0.001**
**Time × Age_{BAS}**	-0.000	0.012	[-0.023, 0.022]	0.994
**Time × Sex**	-0.000	0.023	[-0.046, 0.045]	0.995
**Time × Aβ LI_{BAS}**	0.025	0.012	[0.003, 0.048]	**0.028**

Table S2.4. Summary of the linear mixed effects model within A+T- subsample predicting tau laterality over time with baseline Aβ laterality at Braak meta-ROIs.
The statistical significance of the baseline Aβ laterality and its interaction with time are annotated with **green** if

it reached $p<0.05$ and **red** if it did not. Furthermore, * indicates whether the p-value survived the statistical threshold after Bonferroni correction.

A+T- (n=180 with a total of 452 datapoints)<br>LME: **Tau LI** ~ **time** * (Age$_{BAS}$ + Sex + **Aβ LI$_{BAS}$**) + [time | participant]												
**ROI**	**Braak I-II**				**Braak III-IV**				**Braak V-VI**			
	**β**	**SE**	**95% CI**	**p**	**β**	**SE**	**95% CI**	**p**	**β**	**SE**	**95% CI**	**p**
**Intercept**	-0.014	0.106	[-0.221, 0.193]	0.894	-0.041	0.067	[-0.172, 0.091]	0.545	-0.046	0.076	[-0.195, 0.102]	0.539
**Time**	0.030	0.026	[-0.021, 0.082]	0.250	0.021	0.029	[-0.036, 0.079]	0.471	0.013	0.035	[-0.056, 0.081]	0.713
**Age$_{BAS}$**	-0.013	0.075	[-0.161, 0.134]	0.861	-0.042	0.048	[-0.136, 0.052]	0.378	-0.096	0.054	[-0.201, 0.010]	0.075
**Sex**	-0.023	0.151	[-0.318, 0.273]	0.881	-0.087	0.096	[-0.275, 0.101]	0.363	0.017	0.107	[-0.193, 0.228]	0.871
**Aβ LI$_{BAS}$**	0.067	0.074	[-0.077, 0.212]	**0.363**	0.239	0.048	[0.146, 0.332]	**<0.001***	0.202	0.053	[0.098, 0.306]	**<0.001***
**Time × Age$_{BAS}$**	-0.026	0.019	[-0.063, 0.010]	0.158	-0.020	0.021	[-0.061, 0.020]	0.327	-0.018	0.025	[-0.066, 0.030]	0.464
**Time × Sex**	-0.041	0.037	[-0.113, 0.032]	0.269	0.058	0.041	[-0.023, 0.139]	0.162	0.036	0.049	[-0.060, 0.131]	0.464
**Time × Aβ LI$_{BAS}$**	0.054	0.019	[0.017, 0.091]	**0.004***	0.078	0.021	[0.038, 0.119]	**<0.001***	0.048	0.025	[-0.000, 0.096]	**0.050**

Table S2.5. Summary of the linear mixed effects model within A+T+ subsample predicting tau laterality over time with baseline Aβ laterality at Braak meta-ROIs.
The statistical significance of the baseline Aβ laterality and its interaction with time are annotated with **green** if it reached $p<0.05$ and **red** if it did not. Furthermore, * indicates whether the p-value survived the statistical threshold after Bonferroni correction.

A+T+ (n=109 with a total of 255 datapoints)<br>LME: **Tau LI** ~ **time** * (Age$_{BAS}$ + Sex + **Aβ LI$_{BAS}$**) + [time | participant]												
**ROI**	**Braak I-II**				**Braak III-IV**				**Braak V-VI**			
	**β**	**SE**	**95% CI**	**p**	**β**	**SE**	**95% CI**	**p**	**β**	**SE**	**95% CI**	**p**
**Intercept**	-0.047	0.121	[-0.284, 0.190]	0.700	-0.091	0.074	[-0.237, 0.055]	0.223	-0.054	0.074	[-0.199, 0.091]	0.466
**Time**	-0.010	0.036	[-0.080, 0.060]	0.778	0.002	0.020	[-0.037, 0.041]	0.915	-0.017	0.027	[-0.069, 0.035]	0.526
**Age$_{BAS}$**	-0.058	0.089	[-0.232, 0.116]	0.511	-0.030	0.054	[-0.137, 0.076]	0.576	0.015	0.054	[-0.091, 0.122]	0.781
**Sex**	0.041	0.175	[-0.303, 0.384]	0.817	0.118	0.108	[-0.094, 0.330]	0.277	0.068	0.107	[-0.143, 0.278]	0.528
**Aβ LI$_{BAS}$**	0.280	0.088	[0.107, 0.453]	**0.002***	0.688	0.053	[0.583, 0.792]	**<0.001***	0.584	0.053	[0.481, 0.688]	**<0.001***
**Time × Age$_{BAS}$**	0.009	0.026	[-0.042, 0.061]	0.719	0.003	0.015	[-0.025, 0.032]	0.828	0.003	0.020	[-0.035, 0.042]	0.871

**Time × Sex**	0.063	0.054	[-0.043, 0.169]	0.243	-0.009	0.030	[-0.069, 0.050]	0.754	-0.009	0.039	[-0.087, 0.068]	0.810
**Time × Aβ LI$_{BAS}$**	0.024	0.026	[-0.028, 0.076]	**0.369**	0.004	0.015	[-0.025, 0.034]	**0.770**	0.033	0.019	[-0.005, 0.071]	**0.090**

Table S2.6. Summary of the linear mixed effects model within A+T- subsample who stay A+T- throughout follow-up predicting tau laterality over time with baseline Aβ laterality at Braak meta-ROIs. The statistical significance of the baseline Aβ laterality and its interaction with time are annotated with **green** if it reached $p<0.05$ and **red** if it did not. Furthermore, * indicates whether the p-value survived the statistical threshold after Bonferroni correction.

A+T- to A+T- (n=142 with a total of 347 datapoints)<br>LME: **Tau LI** ~ **time** * (Age$_{BAS}$ + Sex + **Aβ LI$_{BAS}$**) + [time | participant]												
**ROI**	**Braak I-II**				**Braak III-IV**				**Braak V-VI**			
	**β**	**SE**	**95% CI**	**p**	**β**	**SE**	**95% CI**	**p**	**β**	**SE**	**95% CI**	**p**
**Intercept**	0.010	0.121	[-0.227, 0.246]	0.936	0.007	0.093	[-0.175, 0.189]	0.936	-0.048	0.097	[-0.238, 0.141]	0.616
**Time**	0.036	0.030	[-0.023, 0.096]	0.230	0.044	0.038	[-0.031, 0.119]	0.246	0.034	0.038	[-0.039, 0.108]	0.363
**Age$_{BAS}$**	0.014	0.087	[-0.157, 0.185]	0.872	-0.074	0.067	[-0.205, 0.058]	0.271	-0.117	0.070	[-0.254, 0.020]	0.093
**Sex**	-0.055	0.174	[-0.396, 0.287]	0.753	-0.115	0.134	[-0.377, 0.147]	0.389	0.001	0.139	[-0.272, 0.274]	0.995
**Aβ LI$_{BAS}$**	0.039	0.086	[-0.129, 0.206]	**0.651**	0.263	0.066	[0.134, 0.393]	**<0.001***	0.171	0.069	[0.035, 0.307]	**0.013***
**Time × Age$_{BAS}$**	-0.014	0.022	[-0.056, 0.029]	0.529	0.023	0.027	[-0.030, 0.076]	0.401	0.015	0.026	[-0.037, 0.067]	0.568
**Time × Sex**	-0.045	0.043	[-0.129, 0.039]	0.298	0.002	0.054	[-0.104, 0.109]	0.964	0.007	0.053	[-0.096, 0.109]	0.902
**Time × Aβ LI$_{BAS}$**	0.076	0.022	[0.033, 0.119]	**0.001***	0.034	0.027	[-0.018, 0.087]	**0.199**	-0.001	0.026	[-0.051, 0.050]	**0.979**

Table S2.7. Summary of the linear mixed effects model within A+T- subsample who progress to A+T+ during follow-up predicting tau laterality over time with baseline Aβ laterality at Braak meta-ROIs. The statistical significance of the baseline Aβ laterality and its interaction with time are annotated with **green** if it reached $p<0.05$ and **red** if it did not. Furthermore, * indicates whether the p-value survived the statistical threshold after Bonferroni correction.

A+T- to A+T+ (n=38 with a total of 105 datapoints)<br>LME: **Tau LI** ~ **time** * (Age$_{BAS}$ + Sex + **Aβ LI$_{BAS}$**) + [time | participant]												
**ROI**	**Braak I-II**				**Braak III-IV**				**Braak V-VI**			
	**β**	**SE**	**95% CI**	**p**	**β**	**SE**	**95% CI**	**p**	**β**	**SE**	**95% CI**	**p**
**Intercept**	-0.073	0.243	[-0.550, 0.405]	0.766	-0.107	0.141	[-0.385, 0.170]	0.447	-0.058	0.131	[-0.315, 0.199]	0.658
**Time**	-0.021	0.057	[-0.133, 0.092]	0.721	-0.015	0.041	[-0.096, 0.065]	0.713	-0.048	0.061	[-0.167, 0.070]	0.424

**Age$_{BAS}$**	-0.136	0.166	[-0.461, 0.189]	0.411	0.028	0.099	[-0.166, 0.222]	0.776	-0.050	0.090	[-0.226, 0.126]	0.579
**Sex**	0.053	0.335	[-0.604, 0.710]	0.875	-0.049	0.194	[-0.431, 0.332]	0.799	0.078	0.179	[-0.273, 0.428]	0.664
**Aβ LI$_{BAS}$**	0.184	0.166	[-0.141, 0.509]	**0.268**	0.316	0.099	[0.121, 0.511]	**0.001***	0.303	0.087	[0.132, 0.475]	**0.001***
**Time × Age$_{BAS}$**	-0.077	0.038	[-0.151, -0.003]	0.041	-0.096	0.029	[-0.153, -0.040]	0.001	-0.082	0.042	[-0.165, -0.000]	0.050
**Time × Sex**	0.000	0.075	[-0.147, 0.148]	0.998	0.143	0.055	[0.035, 0.251]	0.009	0.099	0.082	[-0.061, 0.260]	0.224
**Time × Aβ LI$_{BAS}$**	-0.003	0.037	[-0.075, 0.070]	**0.942**	0.131	0.029	[0.075, 0.188]	**<0.001***	0.144	0.043	[0.060, 0.227]	**0.001***

Longitudinal association between baseline Aβ/tau laterality and changes over time in mPACC scores

Table S2.8. Summary of the linear mixed effects models predicting mPACC scores over time with pathological asymmetry across the Braak meta-ROIs: (1) baseline tau laterality as predictor; (2) baseline tau laterality as predictor after adjusting for tau load; (3) baseline Aβ laterality as predictor.
The statistical significance of the interaction between time and baseline Aβ/tau laterality is annotated with **green** if it reached p<0.05 and **red** if it did not. Furthermore, * indicates whether the p-value survived the statistical threshold after Bonferroni correction.

A+ (n=259 with a total of 606 datapoints)												
LME #1: **mPACC** ~ **time** * (Age$_{BAS}$ + Sex + **Tau LI$_{BAS}$**) + [time | participant]												
**ROI**	**Braak I-II**				**Braak III-IV**				**Braak V-VI**			
	**β**	**SE**	**95% CI**	**p**	**β**	**SE**	**95% CI**	**p**	**β**	**SE**	**95% CI**	**p**
**Intercept**	0.139	0.060	[0.020, 0.257]	0.022	0.141	0.058	[0.029, 0.254]	0.014	0.157	0.057	[0.045, 0.270]	0.006
**Time**	-0.160	0.029	[-0.216, -0.104]	<0.001	-0.162	0.026	[-0.214, -0.111]	<0.001	-0.157	0.025	[-0.205, -0.108]	<0.001
**Age$_{BAS}$**	-0.122	0.043	[-0.206, -0.038]	0.004	-0.130	0.041	[-0.209, -0.050]	0.001	-0.147	0.041	[-0.227, -0.067]	<0.001
**Sex**	0.170	0.086	[0.002, 0.338]	0.048	0.187	0.082	[0.026, 0.347]	0.022	0.153	0.082	[-0.007, 0.313]	0.060
**Tau LI$_{BAS}$**	0.034	0.043	[-0.051, 0.119]	0.432	-0.202	0.040	[-0.280, -0.124]	<0.001	-0.206	0.040	[-0.284, -0.128]	<0.001
**Time × Age$_{BAS}$**	-0.015	0.021	[-0.056, 0.025]	0.462	-0.022	0.019	[-0.059, 0.015]	0.243	-0.035	0.018	[-0.070, 0.000]	0.053
**Time × Sex**	-0.021	0.041	[-0.101, 0.060]	0.616	-0.017	0.037	[-0.090, 0.056]	0.643	-0.031	0.035	[-0.100, 0.038]	0.372
**Time × Tau LI$_{BAS}$**	-0.036	0.020	[-0.076, 0.004]	**0.076**	-0.132	0.019	[-0.171, -0.094]	**<0.001***	-0.157	0.019	[-0.194, -0.121]	**<0.001***
LME #2: **mPACC** ~ **time** * (Age$_{BAS}$ + Sex + Tau load$_{BAS}$ + **Tau LI$_{BAS}$**) + [time | participant]												
**ROI**	**Braak I-II**				**Braak III-IV**				**Braak V-VI**			

	β	SE	95% CI	p	β	SE	95% CI	p	β	SE	95% CI	p
**Intercept**	0.154	0.057	[0.043, 0.266]	0.007	0.157	0.056	[0.047, 0.266]	0.005	0.163	0.057	[0.051, 0.275]	0.004
**Time**	-0.153	0.026	[-0.204, -0.101]	<0.001	-0.157	0.024	[-0.203, -0.110]	<0.001	-0.156	0.024	[-0.203, -0.108]	<0.001
**Age$_{BAS}$**	-0.133	0.040	[-0.212, -0.054]	0.001	-0.135	0.039	[-0.213, -0.058]	0.001	-0.150	0.041	[-0.230, -0.071]	<0.001
**Sex**	0.148	0.081	[-0.010, 0.307]	0.067	0.166	0.079	[0.011, 0.322]	0.036	0.148	0.081	[-0.011, 0.307]	0.068
**Tau load$_{BAS}$**	-0.241	0.041	[-0.321, -0.161]	<0.001	-0.218	0.057	[-0.330, -0.106]	<0.001	-0.093	0.055	[-0.200, 0.014]	0.088
**Tau LI$_{BAS}$**	0.083	0.042	[0.001, 0.164]	0.046	-0.043	0.057	[-0.154, 0.068]	0.449	-0.143	0.054	[-0.250, -0.037]	0.008
**Time × Age$_{BAS}$**	-0.020	0.019	[-0.057, 0.017]	0.282	-0.029	0.017	[-0.063, 0.004]	0.088	-0.036	0.018	[-0.071, -0.002]	0.039
**Time × Sex**	-0.033	0.037	[-0.106, 0.040]	0.373	-0.034	0.034	[-0.100, 0.032]	0.311	-0.035	0.034	[-0.102, 0.032]	0.306
**Time × Tau load$_{BAS}$**	-0.117	0.019	[-0.155, -0.080]	<0.001	-0.166	0.025	[-0.216, -0.117]	<0.001	-0.077	0.025	[-0.125, -0.029]	0.002
**Time × Tau LI$_{BAS}$**	-0.010	0.019	[-0.047, 0.028]	**0.611**	-0.013	0.025	[-0.062, 0.037]	**0.615**	-0.104	0.025	[-0.153, -0.055]	**<0.001***
LME #3: **mPACC ~ time** * (Age$_{BAS}$ + Sex + **Aβ LI$_{BAS}$**) + [time | participant]												
**ROI**	**Braak I-II**				**Braak III-IV**				**Braak V-VI**			
	**β**	**SE**	**95% CI**	**p**	**β**	**SE**	**95% CI**	**p**	**β**	**SE**	**95% CI**	**p**
**Intercept**	0.142	0.060	[0.023, 0.260]	0.019	0.142	0.060	[0.025, 0.259]	0.017	0.145	0.060	[0.027, 0.263]	0.016
**Time**	-0.161	0.029	[-0.217, -0.105]	<0.001	-0.162	0.029	[-0.218, -0.106]	<0.001	-0.161	0.029	[-0.218, -0.105]	<0.001
**Age$_{BAS}$**	-0.117	0.043	[-0.201, -0.033]	0.006	-0.123	0.042	[-0.206, -0.041]	0.003	-0.120	0.042	[-0.204, -0.037]	0.004
**Sex**	0.165	0.086	[-0.003, 0.334]	0.054	0.162	0.085	[-0.004, 0.328]	0.056	0.155	0.086	[-0.013, 0.322]	0.070
**Aβ LI$_{BAS}$**	-0.045	0.043	[-0.129, 0.039]	0.297	0.114	0.043	[0.031, 0.197]	0.007	0.089	0.042	[0.006, 0.172]	0.035
**Time × Age$_{BAS}$**	-0.016	0.021	[-0.057, 0.024]	0.427	-0.018	0.021	[-0.058, 0.023]	0.392	-0.017	0.021	[-0.058, 0.023]	0.397
**Time × Sex**	-0.019	0.041	[-0.099, 0.061]	0.639	-0.019	0.041	[-0.099, 0.062]	0.649	-0.020	0.041	[-0.100, 0.060]	0.626
**Time × Aβ LI$_{BAS}$**	-0.012	0.021	[-0.052, 0.029]	**0.572**	0.014	0.020	[-0.026, 0.054]	**0.507**	0.012	0.020	[-0.029, 0.052]	**0.573**

Replication in independent cohorts

Table S2.9. Demographics of the external cohorts.
Categorical variables have been presented as 'count (%)', normally distributed continuous variables as 'mean (SD)' and non-normally distributed variables as 'median [interquartile range]'. OASIS-3, Open Access Series of Imaging Studies; A4, Anti-Amyloid Treatment in Asymptomatic Alzheimer's Disease; ADNI, Alzheimer's Disease Neuroimaging Initiative; M, male; F, female; CU, cognitively unimpaired; CI, cognitively impaired; SUVR, standardized uptake value ratio; Aβ, amyloid-beta.

		OASIS-3 (A+T+; n=46)	A4 (A+T+; n=55)	ADNI (A+T+; n=133)
**Age, years**		74.61 (6.77)	72.65 (5.06)	72.39 (6.72)
**Sex**	**M**	21 (46%)	21 (38%)	56 (42%)
	**F**	25 (54%)	34 (62%)	77 (58%)
**Education, years**		15.78 (2.60)	16.60 (2.66)	15.69 (2.40)
**Diagnosis**	**CU**	15 (33%)	55 (100%)	23 (17%)
	**CI**	31 (67%)	0 (0%)	110 (83%)
**Temporal tau, SUVR**		1.57 [1.45,1.85]	1.38 [1.34,1.46]	1.57 [1.39,1.82]
**Global Aβ, SUVR**		1.40 [1.27,1.59]	1.37 [1.27,1.46]	1.42 [1.32,1.53]

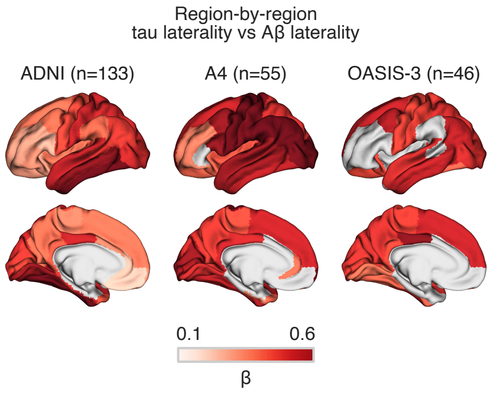

Figure S2.9. Region-by-region associations between Aβ laterality and tau laterality in external cohorts. Aβ, amyloid-beta; OASIS-3, Open Access Series of Imaging Studies; A4, Anti-Amyloid Treatment in Asymptomatic Alzheimer's Disease; ADNI, Alzheimer's Disease Neuroimaging Initiative.

Partial volume corrected PET

We performed cross-sectional and longitudinal sensitivity analyses using partial volume corrected PET SUVR values to investigate if the association between laterality of Aβ and tau distribution could be related to this methodological choice. Cross-sectionally, the analysis showed a similarly strong association between Aβ laterality and tau laterality globally (Fig. S2.10a; $\beta=0.648$, $p<0.001$) and across all Braak meta-ROIs (Fig. S2.10b; Braak I-II: $\beta=0.178$,

p_{Bonf}=0.016; Braak III-IV: β=0.728, p_{Bonf}<0.001; Braak V-VI: β=0.590, p_{Bonf}<0.001). One outlier was removed from the analysis based on the 4 SD difference from the mean. Moreover, the longitudinal sensitivity analysis provided similar results to the main results with the full A+ sample where higher baseline Aβ laterality was predictive of changes over time in tau laterality (Fig. S2.11a; β=0.041, 95%CI=[0.021; 0.060], p<0.001). Similarly, A+T- group showed significant interaction effect only in Braak I-II (Fig. S2.11b; β=0.078, 95%CI=[0.027; 0.129], p_{Bonf}=0.009) and Braak III-IV (β=0.085, 95%CI=[0.041; 0.129], p_{Bonf}<0.001). However, A+T+ group exhibited significant effect on baseline Aβ laterality on tau laterality over time in Braak V-VI (Fig. S2.11c; β=0.064, 95%CI=[0.034; 0.094], p_{Bonf}<0.001), which we did not identify in the main findings. For individuals who stayed A+T- throughout their follow-up, we detected a significant interaction effect only in Braak I-II (Fig. S2.11d; β=0.113, 95%CI=[0.050; 0.175], p_{Bonf}=0.001), which was in accordance with the main findings, but for people who converted to A+T+ we did not find any significant association in any of the Braak meta-ROIs in contrast to our main findings – only trending level significance in Braak III-IV (β=0.081, 95%CI=[0.015; 0.147], p_{Bonf}=0.051) and Braak V-VI (Fig. S2.11e; β=0.093, 95%CI=[0.014; 0.171], p_{Bonf}=0.061).

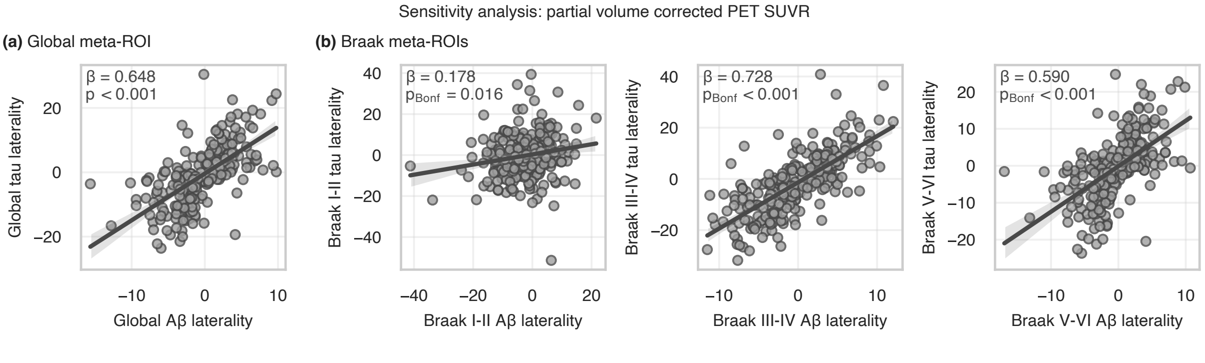

Figure S2.10. Cross-sectional association between Aβ and tau laterality with using partial volume corrected SUVR values for calculating laterality: (a) at global meta-ROI; (b) at Braak meta-ROIs.
All plots show regression lines with 95% confidence intervals, with statistical annotations indicating the standardized effect size and significance level of Aβ laterality as a predictor of tau laterality in ordinary least squares multiple linear regression models (tau laterality ~ age + sex + Aβ laterality). P-values were Bonferroni-corrected (×3) and annotated. Aβ, amyloid-beta; SUVR, standardised uptake value ratio.

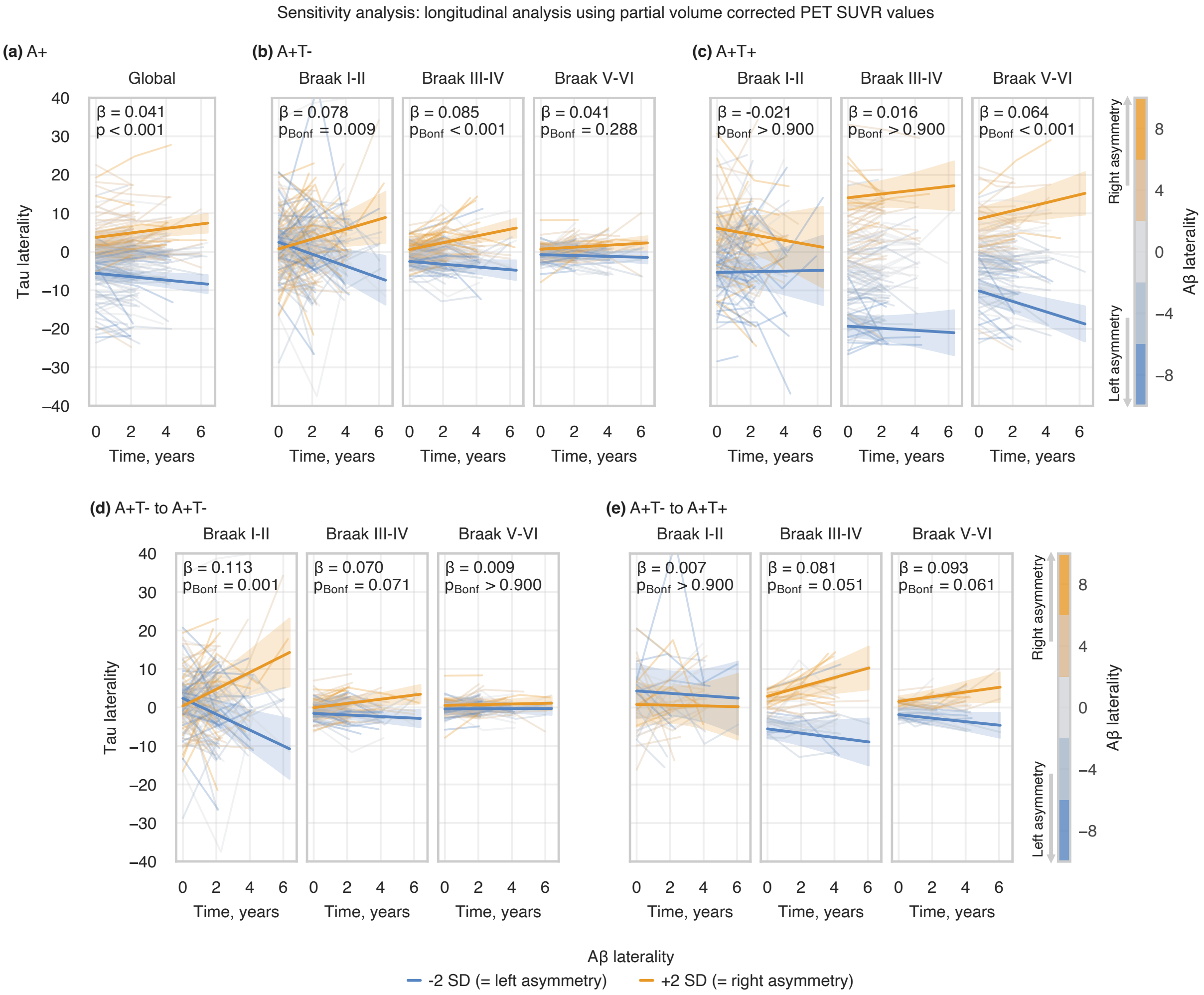

Figure S2.11. Longitudinal analysis of the association between baseline Aβ laterality and changes over time in tau laterality at Braak meta-ROIs using partial volume corrected SUVR value for calculating laterality: (a) whole A+ sample at global meta-ROI (i.e., whole-brain for each hemisphere); (b) A+T- subsample at Braak meta-ROIs; (c) A+T+ subsample at Braak meta-ROIs; (d) A+T- subsample who stay A+T- throughout their follow-up; (e) A+T- subsample who progress to A+T+ during their follow-up.
The statistical analyses were performed using linear mixed effects models with random intercepts and slopes for time and participants (tau LI ~ time * ($age_{baseline}$ + sex + Aβ $LI_{baseline}$) + [1 + time | participant]), with p-values Bonferroni-corrected for the number of meta-ROIs tested. The statistical annotations indicate the standardized effect size and significance level of the interaction between time and baseline Aβ laterality on tau laterality. For visualization, regression lines represent the modeled mean tau laterality with 95% confidence intervals, plotted for LI_{ref}±2 SD of baseline Aβ laterality, where LI_{ref}=0 (i.e., perfect Aβ symmetry). Colorbar indicates baseline Aβ laterality index. Aβ, amyloid-beta; LI, laterality index.

Association of the distribution of Aβ and tau with cerebral blood flow and cortical thickness

To test whether our main cross-sectional finding of a strong association between Aβ and tau distribution was not due to other biological confounders, we tested to what extent Aβ laterality and tau laterality are associated to laterality in cerebral blood flow and cortical thickness.

Cerebral blood flow was estimated using arterial spin labelling (ASL) scans which were acquired on a subset of the sample and its methodology in detail has been described previously.[35] Cortical thickness was measured as the distance from the grey matter/white matter boundary to the corresponding pial surface.[36] Only tau laterality was negatively related to laterality in cerebral blood flow (n=101; β=-0.527, p<0.001), but not Aβ (n=53; β=-0.241, p=0.085) (Fig. S2.12a). Furthermore, the laterality indexes of both pathologies were negatively associated with laterality in cortical thickness, but with greatly stronger effect with tau (n=449; β=-0.629, p<0.001) than Aβ (n=231; β=-0.185, p=0.005) (Fig. S2.12c). Most importantly, the association between the laterality of Aβ and tau distribution was still statistically significant after adjusting for the laterality of either cerebral blood flow (Fig. S2.12b; n=53; β=0.498, p<0.001) or cortical thickness (Fig. S2.12d; n=231; β=0.560, p<0.001).

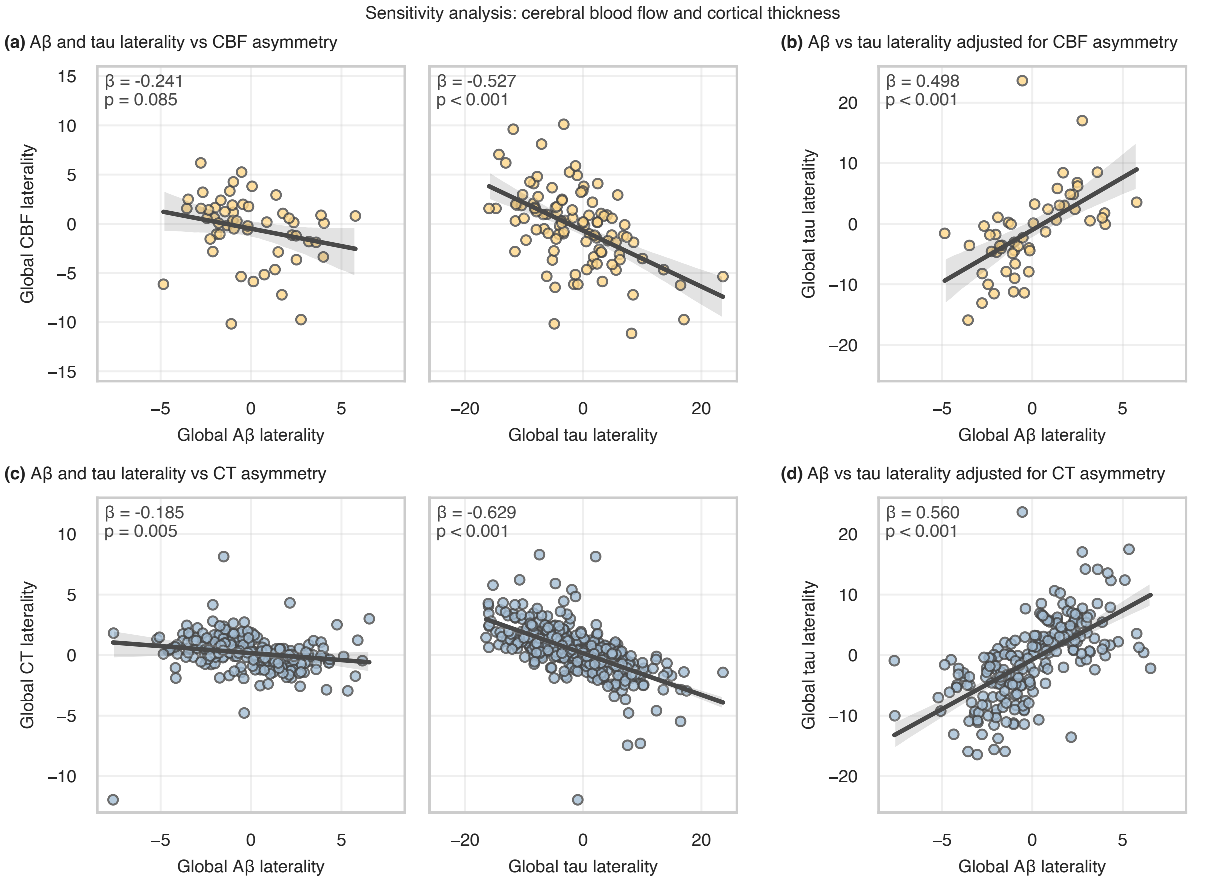

Figure S2.12. Cross-sectional associations between the laterality of both Aβ and tau to the laterality of CBF and CT: (a) between the laterality of Aβ/tau and CBF; (b) between the laterality of Aβ and tau after adjusting for CBF; (c) between the laterality of Aβ/tau and CT; (d) between the laterality of Aβ and tau after adjusting for

CT.
All plots show regression lines with 95% confidence intervals, with statistical annotations indicating the standardized effect size and significance level of a predictor (i.e., x-axis) in ordinary least squares multiple linear regression models (e.g., global CBF laterality ~ age + sex + Aβ laterality). P-values were Bonferroni-corrected (×3) and annotated. Aβ, amyloid-beta; CBF, cerebral blood flow; CT, cortical thickness.

Difference in estimated Aβ onset between hemispheres between the groups

Time difference in Aβ onset between hemispheres was computed using the sampled iterative local approximation (SILA) algorithm[34] to investigate inter-hemispheric difference in Aβ accumulation across the groups defined based on tau laterality index. The SILA algorithm was applied to the full longitudinal BioFINDER-2 cohort to model estimated years until/since Aβ onset for each hemisphere for each meta-ROI. These unilateral values were subtracted within each meta-ROI to obtain the estimated time difference of Aβ onset between hemispheres. For the comparison of the estimated hemispheric time difference in Aβ onset between the groups, OLS multiple linear regressions (OLS: estimated Aβ onset difference ~ age + sex + group) were used with the significance level ($p<0.05$) Bonferroni-corrected for the number of group comparisons performed (3 comparisons: LA vs S, RA vs S, and LA vs RA).

We found that the difference in estimated onset of global Aβ pathology between hemispheres was significantly higher in the asymmetric groups compared to the symmetric group with a median difference in the left asymmetric of 1.7 years ($t=3.123$, $p=0.006$) and of 2.5 years in the right asymmetric ($t=4.137$, $p<0.001$), compared to 1.3 years in the symmetric group, which was similar across other meta-ROIs (Fig. S2.13).

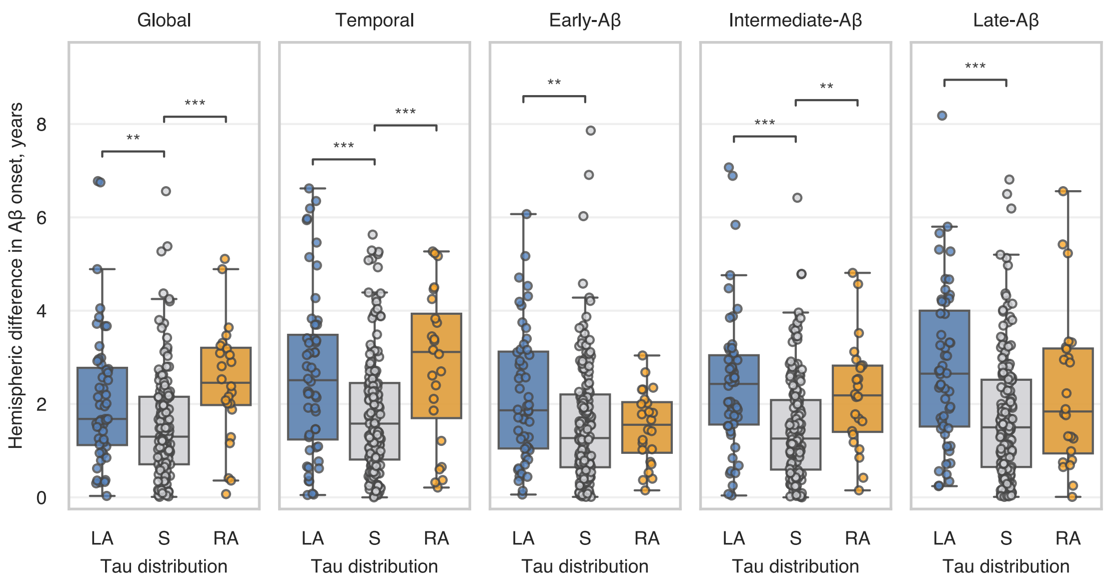

Figure S2.13. Comparison of the SILA-based estimation of time difference in Aβ onset between hemispheres across meta-ROIs.
Boxplots represent estimated hemispheric difference in Aβ onset time across the three tau asymmetry groups, where the groups were statistically compared using ordinary least squares multiple linear regression models, with the significance levels Bonferroni-corrected for the number of group comparisons. The horizontal line within each box indicates the median, while the lower and upper box edges denote the first and third quartiles, respectively. Whiskers extend to 1.5 times the interquartile range, and dots represent individual data points. Aβ, amyloid-beta; SILA, sampled iterative local approximation algorithm; LA, left tau asymmetric; S, tau symmetric; RA, right tau asymmetric; **, p<0.01; ***, p<0.001.

Combining connectivity and Aβ laterality to explain tau laterality

To evaluate whether region-specific tau laterality is best explained by a combination of connectivity and Aβ asymmetry, we performed additional analyses comparing two OLS multiple linear regression models: (1) base model: absolute tau laterality index (LI) ~ Aβ LI + age + sex; (2) extended model: base model + homotopic connectivity (FC or SC). For each of the 36 regions, we compared models using F-tests on residual variance, with FDR correction. Across all regions, adding functional connectivity did not improve model fit (ΔF: min=0.840, mean=0.996, max=1.329; all $p_{FDR}>0.7$). Similarly, structural connectivity provided no significant improvement (ΔF: min=0.824, mean=1.006, max=1.138; all $p_{FDR}>0.6$). Notably, connectivity measures themselves were never significant predictors in any of the models (all $p_{FDR}>0.05$). These results align with our earlier findings: while Aβ asymmetry strongly

correlates with tau lateralisation, inter-hemispheric connectivity seems not to affect this relationship.

Association between tau and Aβ laterality and longitudinal change in tau burden

To assess whether asymmetry in tau or Aβ pathology predicts faster tau accumulation, we fitted two LME models in the longitudinal Aβ-positive cohort: for tau laterality (LME: tau load ~ time * ($age_{baseline}$ + sex + Aβ $load_{baseline}$ + tau $LI_{baseline}$) + [1 + time | participant]) and for Aβ laterality (LME: tau load ~ time * ($age_{baseline}$ + sex + Aβ $load_{baseline}$ + Aβ $LI_{baseline}$) + [1 + time | participant]), with Bonferroni correction applied to p-values for multiple comparisons across the meta-ROIs analysed. We found that higher baseline absolute tau laterality predicted faster tau burden increases in Braak III-IV (β=0.085, p_{Bonf}<0.001) and Braak V-VI (β=0.114, p_{Bonf}<0.001) regions but not in Braak I-II (Fig. S2.14a). In contrast, Aβ laterality did not display any significant effect on longitudinal change in tau burden in any of the meta-ROIs (all p_{Bonf}>0.200; Fig. S2.14b). These results indicate that tau lateralisation is linked to accelerated pathological disease progression in affected regions, a relationship independent of Aβ asymmetry.

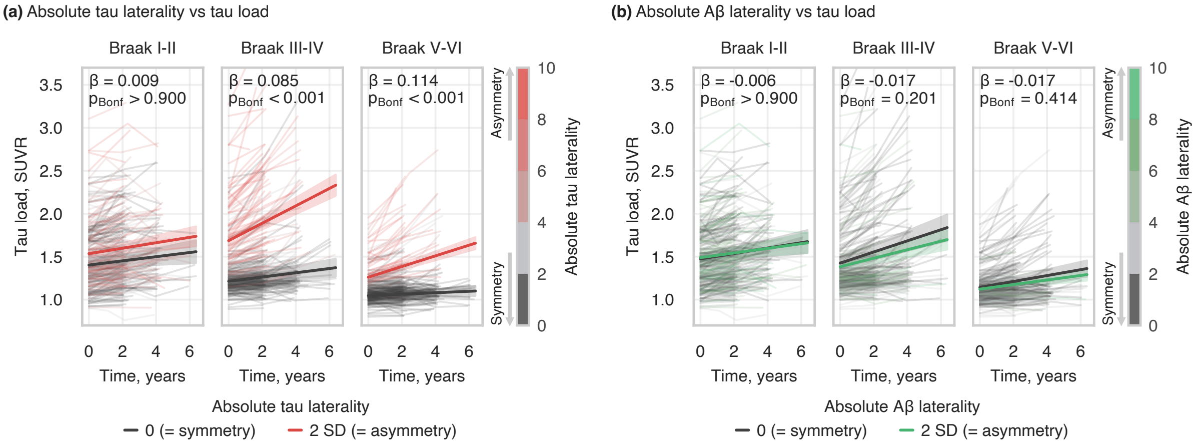

Figure S2.14. Longitudinal analysis of the association between pathological asymmetry and bilateral tau load across meta-ROIs within the A+ sample: (a) association between absolute tau laterality at baseline and tau load; (b) association between absolute Aβ laterality at baseline and tau load.

The statistical analyses were performed using linear mixed effects models with random intercepts and slopes for time and participants, with p-values Bonferroni-corrected for the number of meta-ROIs tested. The statistical annotations indicate the standardized effect size and significance level of the interaction between time and baseline Aβ/tau laterality on tau load. For visualization, regression lines represent the modeled mean cognitive test score with 95% confidence intervals, plotted for LI_{ref} and LI_{ref}+2 SD of baseline Aβ/tau laterality, where LI_{ref}=0 (i.e., perfect Aβ/tau symmetry). Aβ, amyloid-beta; SUVR, standardized uptake value ratio.

Directionality of tau and Aβ laterality and cognition

To assess whether left- or right-predominant pathological asymmetry differentially impacts cognition, we investigated the effect of baseline tau and Aβ laterality on mPACC scores using the non-absolute laterality indices (more positive values = more right asymmetry; more negative values = more left asymmetry). The direction of the tau lateralisation did not lead to different trajectories of mPACC scores in any regions (Fig. S2.15ab). Similarly, directionality of Aβ laterality had no effect on cognition (Fig. S2.15c).

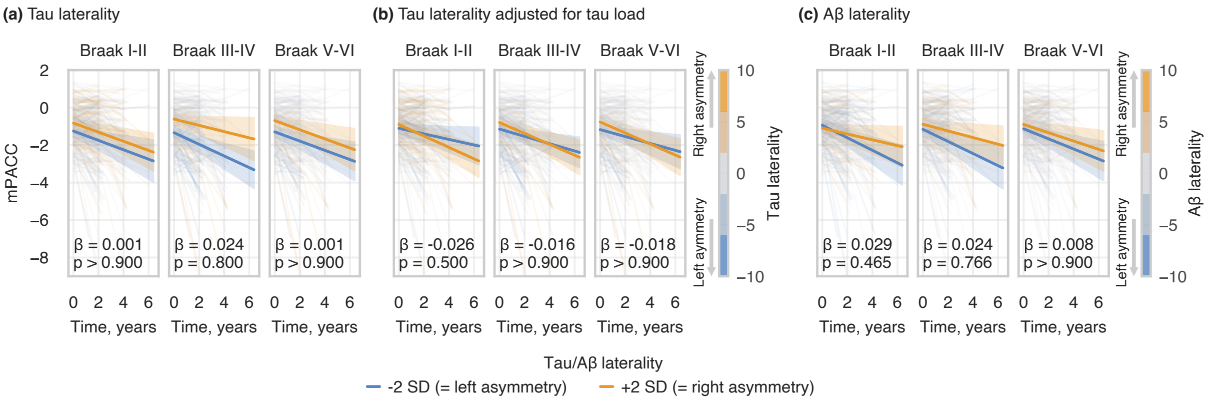

Figure S2.15. Longitudinal analysis within A+ sample over Braak meta-ROIs predicting mPACC score over time with: (a) Baseline tau laterality; (b) Baseline tau laterality after adjusting for tau load; (c) Baseline Aβ laterality after adjusting for tau laterality, tau load, and Aβ load.
The statistical analyses were performed using linear mixed effects models with random intercepts and slopes for time and participants (e.g., model depicted in panel A: mPACC ~ time * ($age_{baseline}$ + sex + tau $LI_{baseline}$) + [1 + time | participant]). The statistical annotations indicate the standardized effect size and significance level of the interaction between time and baseline Aβ/tau laterality on cognitive test score, with p-values Bonferroni-corrected for the number of meta-ROIs tested in each model. For visualization, regression lines represent the modeled mean cognitive test score with 95% confidence intervals, plotted for LI_{ref}±2 SD of baseline Aβ/tau laterality, where LI_{ref}=0 (i.e., perfect Aβ/tau symmetry). Aβ, amyloid-beta; mPACC, modified Preclinical Alzheimer Cognitive Composite; LI, laterality index.

Supplementary S3 – PET scans of representative cases

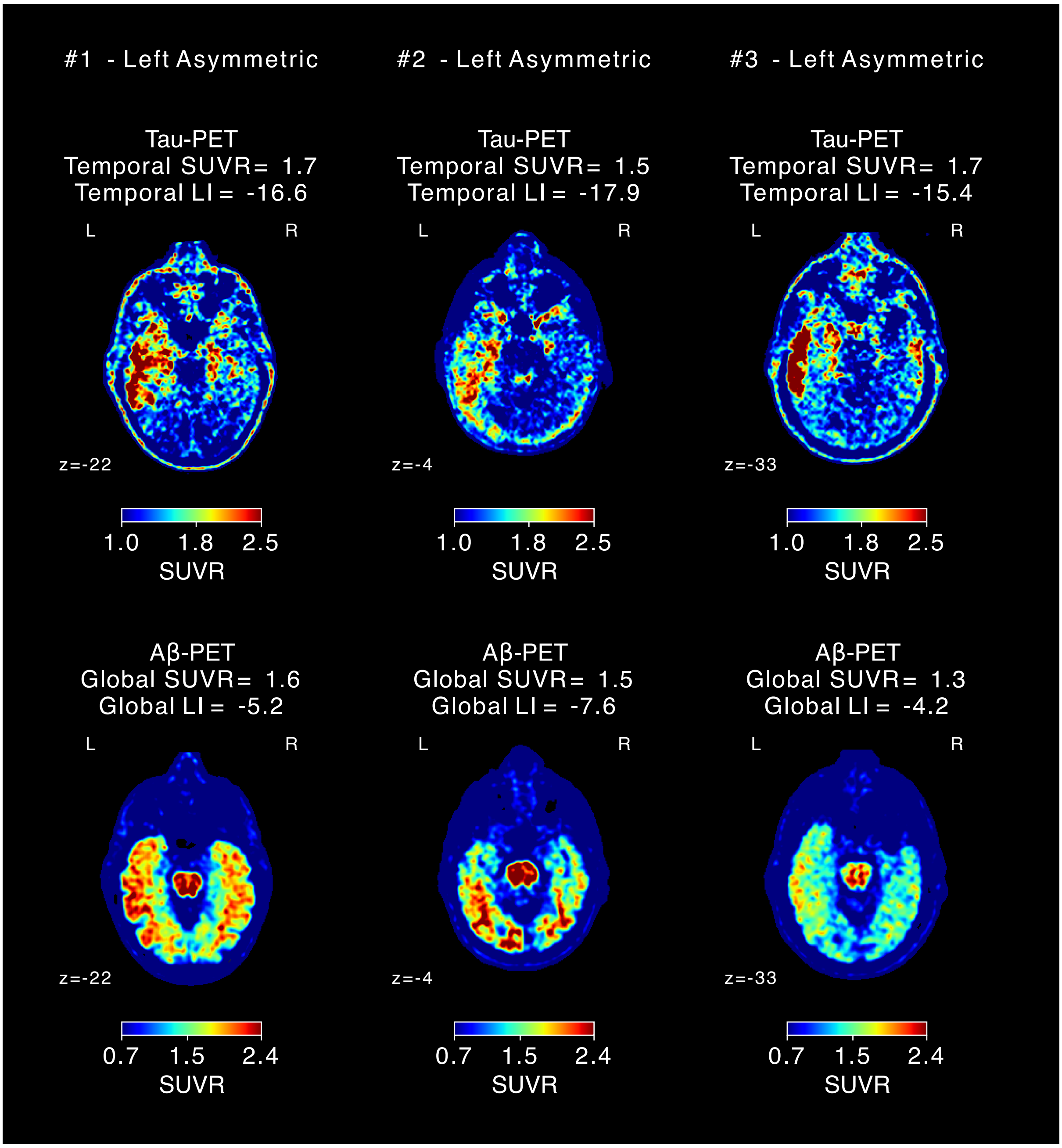

Figure S3.1. Visualisation of tau-PET and Aβ-PET scans for three representative cases with left asymmetric pathological distribution.
SUVR, standardized uptake value ratio; LI, laterality index.

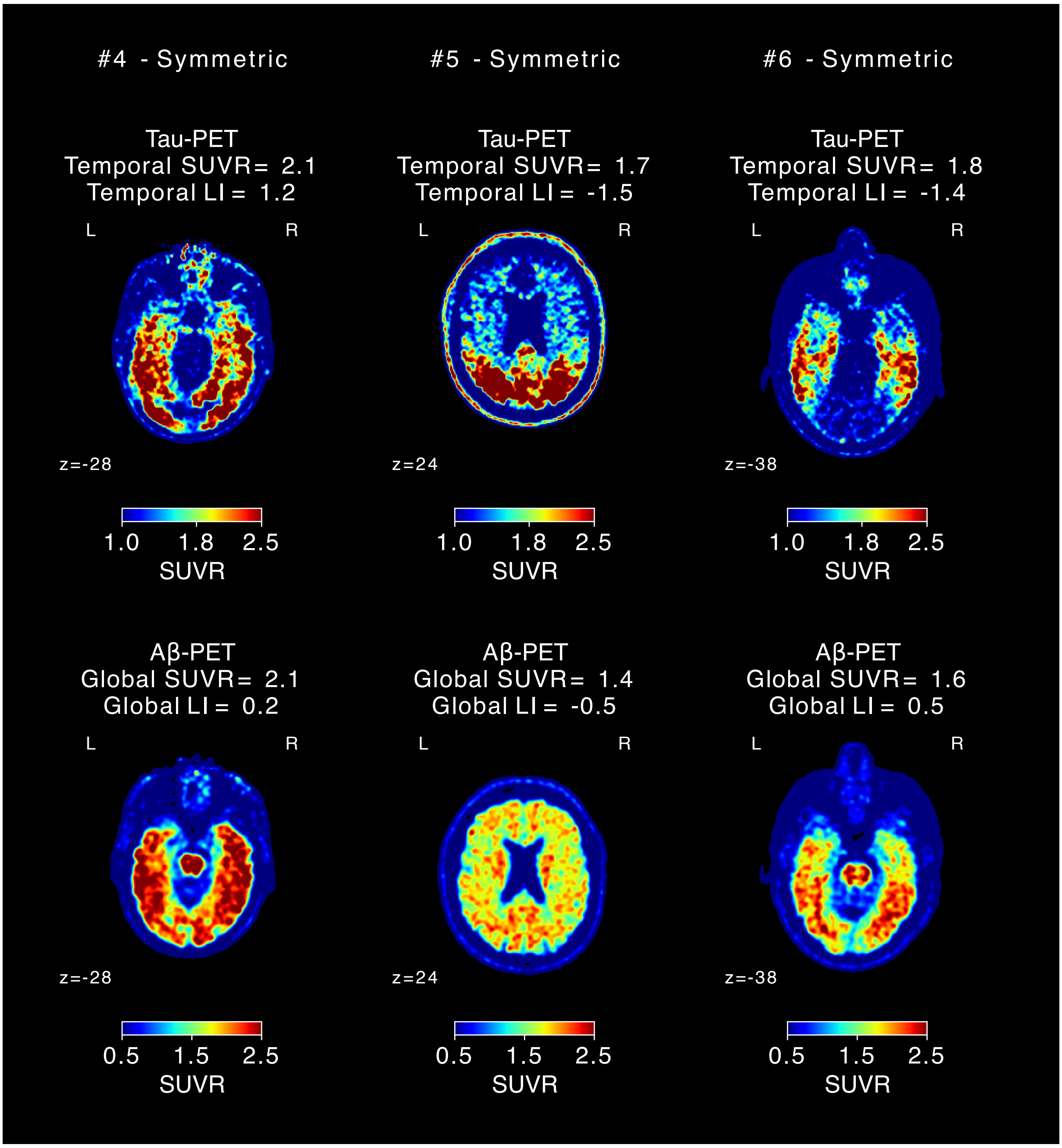

Figure S3.2. Visualisation of tau-PET and Aβ-PET scans for three representative cases with symmetric pathological distribution.
SUVR, standardized uptake value ratio; LI, laterality index.

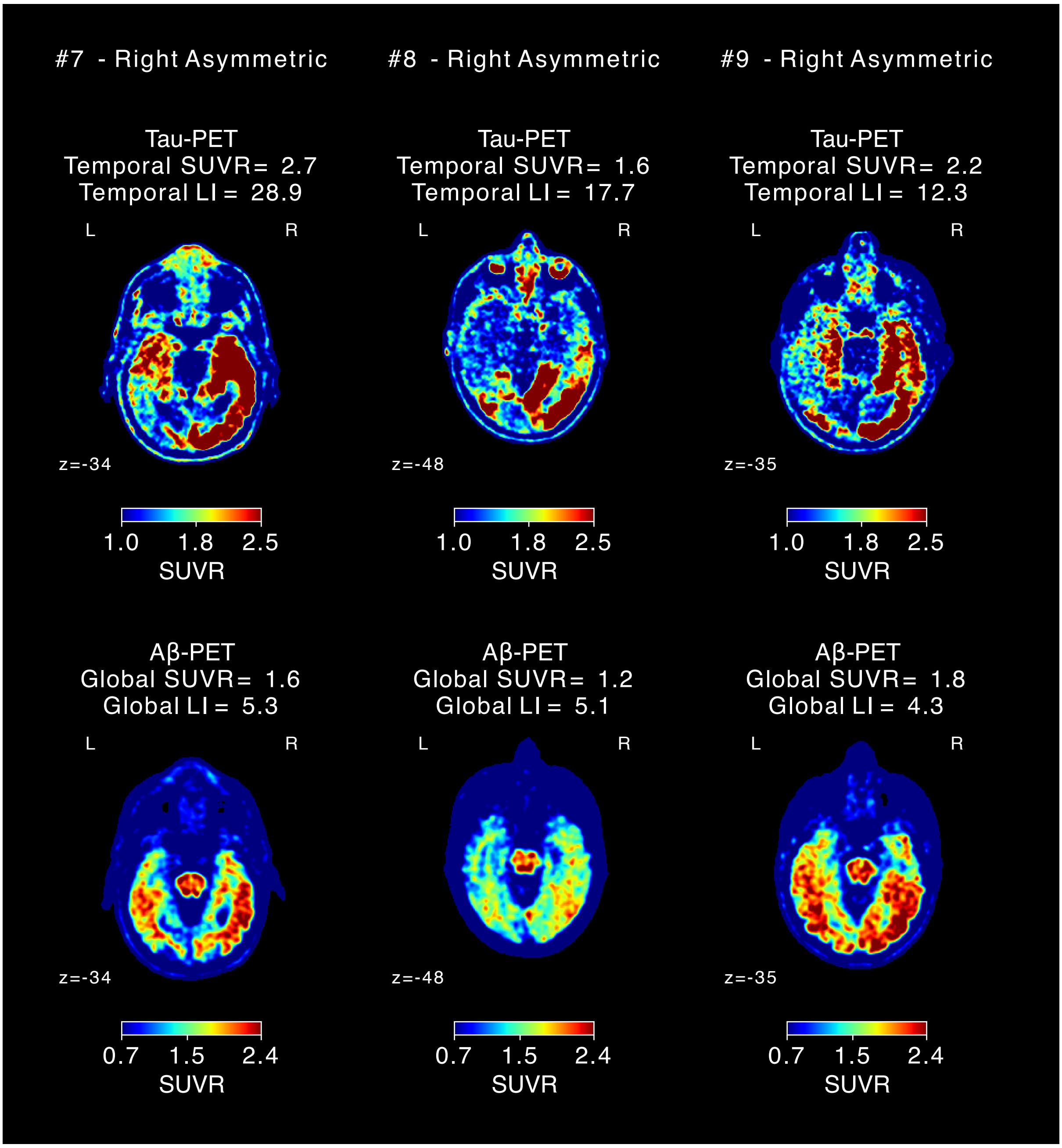

Figure S3.3. Visualisation of tau-PET and Aβ-PET scans for three representative cases with right asymmetric pathological distribution.
SUVR, standardized uptake value ratio; LI, laterality index.

References

1. American Psychiatric Association. *Diagnostic and Statistical Manual of Mental Disorders*. Fifth Edition. American Psychiatric Association; 2013. doi:10.1176/appi.books.9780890425596

2. Palmqvist S, Janelidze S, Quiroz YT, et al. Discriminative Accuracy of Plasma Phospho-tau217 for Alzheimer Disease vs Other Neurodegenerative Disorders. *JAMA*. 2020;324(8):772-781. doi:10.1001/jama.2020.12134

3. Gelb DJ, Oliver E, Gilman S. Diagnostic Criteria for Parkinson Disease. *Arch Neurol*. 1999;56(1):33-39. doi:10.1001/archneur.56.1.33

4. Litvan I, Agid Y, Calne D, et al. Clinical research criteria for the diagnosis of progressive supranuclear palsy (Steele-Richardson-Olszewski syndrome). *Neurology*. 1996;47(1):1-9. doi:10.1212/WNL.47.1.1

5. Gilman S, Wenning GK, Low PA, et al. Second consensus statement on the diagnosis of multiple system atrophy. *Neurology*. 2008;71(9):670-676. doi:10.1212/01.wnl.0000324625.00404.15

6. Armstrong MJ, Litvan I, Lang AE, et al. Criteria for the diagnosis of corticobasal degeneration. *Neurology*. 2013;80(5):496-503. doi:10.1212/WNL.0b013e31827f0fd1

7. Gorno-Tempini ML, Hillis AE, Weintraub S, et al. Classification of primary progressive aphasia and its variants. *Neurology*. 2011;76(11):1006-1014. doi:10.1212/WNL.0b013e31821103e6

8. Braak H, Braak E. Neuropathological stageing of Alzheimer-related changes. *Acta Neuropathol (Berl)*. 1991;82(4):239-259. doi:10.1007/BF00308809

9. Braak H, Alafuzoff I, Arzberger T, Kretzschmar H, Del Tredici K. Staging of Alzheimer disease-associated neurofibrillary pathology using paraffin sections and immunocytochemistry. *Acta Neuropathol (Berl)*. 2006;112(4):389-404. doi:10.1007/s00401-006-0127-z

10. Mattsson N, Palmqvist S, Stomrud E, Vogel J, Hansson O. Staging β-Amyloid Pathology With Amyloid Positron Emission Tomography. *JAMA Neurol*. 2019;76(11):1319-1329. doi:10.1001/jamaneurol.2019.2214

11. Esteban O, Markiewicz CJ, Burns C, et al. nipy/nipype: 1.8.3. Published online July 14, 2022. doi:10.5281/zenodo.6834519

12. Tournier JD, Smith R, Raffelt D, et al. MRtrix3: A fast, flexible and open software framework for medical image processing and visualisation. *NeuroImage*. 2019;202:116137. doi:10.1016/j.neuroimage.2019.116137

13. Jenkinson M, Beckmann CF, Behrens TEJ, Woolrich MW, Smith SM. FSL. *NeuroImage*. 2012;62(2):782-790. doi:10.1016/j.neuroimage.2011.09.015

14. Fischl B. FreeSurfer. *NeuroImage*. 2012;62(2):774-781. doi:10.1016/j.neuroimage.2012.01.021

15. Dhollander T, Raffelt D, Connelly A. Unsupervised 3-tissue response function estimation from single-shell or multi-shell diffusion MR data without a co-registered T1 image. In: *ISMRM Workshop on Breaking the Barriers of Diffusion MRI, 2016, 5.* ; 2016.

16. Dhollander T, Mito R, Raffelt D, Connelly A. Improved white matter response function estimation for 3-tissue constrained spherical deconvolution. In: *Proc Intl Soc Mag Reson Med, 2019, 555.* ; 2019.

17. Tournier JD, Calamante F, Gadian DG, Connelly A. Direct estimation of the fiber orientation density function from diffusion-weighted MRI data using spherical deconvolution. *NeuroImage*. 2004;23(3):1176-1185. doi:10.1016/j.neuroimage.2004.07.037

18. Jeurissen B, Tournier JD, Dhollander T, Connelly A, Sijbers J. Multi-tissue constrained spherical deconvolution for improved analysis of multi-shell diffusion MRI data. *NeuroImage*. 2014;103:411-426. doi:10.1016/j.neuroimage.2014.07.061

19. Smith R, Skoch A, Bajada C, Caspers S, Connelly A. Hybrid Surface-Volume Segmentation for improved Anatomically-Constrained Tractography. In: *Proceedings of the Oganisation for Human Brain Mapping*. ; 2020.

20. Jenkinson M, Smith S. A global optimisation method for robust affine registration of brain images. *Med Image Anal*. 2001;5(2):143-156. doi:10.1016/s1361-8415(01)00036-6

21. Jenkinson M, Bannister P, Brady M, Smith S. Improved optimization for the robust and accurate linear registration and motion correction of brain images. *NeuroImage*. 2002;17(2):825-841. doi:10.1016/s1053-8119(02)91132-8

22. Tournier JD, Calamante F, Connelly A. Improved probabilistic streamlines tractography by 2nd order integration over fibre orientation distributions. In: *Proceedings of the International Society for Magnetic Resonance in Medicine, 2010, 1670.* ; 2010.

23. Smith RE, Tournier JD, Calamante F, Connelly A. Anatomically-constrained tractography: Improved diffusion MRI streamlines tractography through effective use of anatomical information. *NeuroImage*. 2012;62(3):1924-1938. doi:10.1016/j.neuroimage.2012.06.005

24. Smith RE, Tournier JD, Calamante F, Connelly A. SIFT2: Enabling dense quantitative assessment of brain white matter connectivity using streamlines tractography. *NeuroImage*. 2015;119:338-351. doi:10.1016/j.neuroimage.2015.06.092

25. Desikan RS, Ségonne F, Fischl B, et al. An automated labeling system for subdividing the human cerebral cortex on MRI scans into gyral based regions of interest. *NeuroImage*. 2006;31(3):968-980. doi:10.1016/j.neuroimage.2006.01.021

26. Fischl B, Salat DH, Busa E, et al. Whole Brain Segmentation: Automated Labeling of Neuroanatomical Structures in the Human Brain. *Neuron*. 2002;33(3):341-355. doi:10.1016/S0896-6273(02)00569-X

27. Smith RE, Tournier JD, Calamante F, Connelly A. The effects of SIFT on the reproducibility and biological accuracy of the structural connectome. *NeuroImage*. 2015;104:253-265. doi:10.1016/j.neuroimage.2014.10.004

28. Basser PJ, Mattiello J, Lebihan D. Estimation of the Effective Self-Diffusion *Tensor* from the NMR Spin Echo. *J Magn Reson B*. 1994;103(3):247-254. doi:10.1006/jmrb.1994.1037

29. Basser PJ, Mattiello J, LeBihan D. MR diffusion tensor spectroscopy and imaging. *Biophys J*. 1994;66(1):259-267. doi:10.1016/S0006-3495(94)80775-1

30. Wasserthal J, Neher P, Maier-Hein KH. TractSeg - Fast and accurate white matter tract segmentation. *NeuroImage*. 2018;183:239-253. doi:10.1016/j.neuroimage.2018.07.070

31. Abraham A, Pedregosa F, Eickenberg M, et al. Machine learning for neuroimaging with scikit-learn. *Front Neuroinformatics*. 2014;8. doi:10.3389/fninf.2014.00014

32. Friston KJ. Functional and effective connectivity: a review. *Brain Connect*. 2011;1(1):13-36. doi:10.1089/brain.2011.0008

33. Fisher RA. On the" probable error" of a coefficient of correlation deduced from a small sample. Published online 1921.

34. Betthauser TJ, Bilgel M, Koscik RL, et al. Multi-method investigation of factors influencing amyloid onset and impairment in three cohorts. *Brain*. 2022;145(11):4065-4079. doi:10.1093/brain/awac213

35. Ahmadi K, Pereira JB, Berron D, et al. Gray matter hypoperfusion is a late pathological event in the course of Alzheimer's disease. *J Cereb Blood Flow Metab Off J Int Soc Cereb Blood Flow Metab*. 2023;43(4):565-580. doi:10.1177/0271678X221141139

36. Fischl B, Dale AM. Measuring the thickness of the human cerebral cortex from magnetic resonance images. *Proc Natl Acad Sci*. 2000;97(20):11050-11055. doi:10.1073/pnas.200033797

Supplementary Note 1

One of the datasets used in the preparation of this article were obtained from the Alzheimer's Disease Neuroimaging Initiative (ADNI) database (adni.loni.usc.edu). As such, the investigators within the ADNI contributed to the design and implementation of ADNI and/or provided data but did not participate in the analysis or writing of this report. A complete listing of ADNI investigators can be found below.

ACKNOWLEDGEMENT LIST FOR ADNI PUBLICATIONS

The Data and Publications Committee, in keeping with the publication policies adopted by the ADNI Steering Committee, here provide lists for standardized acknowledgement. The list consists of two parts: Infrastructure Investigators and Site Investigators. Infrastructure Investigators represent the names responsible for leadership and infrastructure. Site Investigators represent the names of individuals at each recruiting site. All papers, including methodological papers, should have an acknowledgement list that consists of Infrastructure Investigators plus the FULL list.

I. ADNI 1, GO, 2, 3, 4

Part A: Leadership and Infrastructure

Principal Investigator

Michael Weiner, MD	University of California, San Francisco<br>Northern California Institute for Research and Education

ATRI PI and Director of Coordinating Center Clinical Core

Paul Aisen, MD	University of Southern California
Ronald Petersen, MD, PhD	Mayo Clinic, Rochester (co-PI of of Clinical Core)

Executive Committee

Michael Weiner, MD	University of California, San Francisco
Paul Aisen, MD	University of Southern California
Ronald Petersen, MD, PhD	Mayo Clinic, Rochester
Clifford R. Jack, Jr., MD	Mayo Clinic, Rochester
William Jagust, MD	University of California, Berkeley
Susan Landau, PhD	University of California, Berkeley
Monica Rivera-Mindt, PhD	Fordham University; Mt. Sinai Medical Center
Ozioma Okonkwo, PhD	University of Wisconsin
Leslie M. Shaw, PhD	University of Pennsylvania
Edward B. Lee, MD, PhD	University of Pennsylvania
Arthur W. Toga, PhD	University of California, Los Angeles
Laurel Beckett, PhD	University of California, Davis
Danielle Harvey, PhD	University of California, Davis
Robert C. Green, MD, MPH	Boston University
Andrew J. Saykin, PsyD	Indiana University
Kwangsik Nho, PhD	Indiana University
Richard J. Perrin, MD, PhD	Washington University St. Louis
Duygu Tosun, PhD	University of California, San Francisco

ADNI 4 Private Partner Scientific Board (PPSB) Convened by Alzheimer's Association

Pallavi Sachdev, PhD	Eisai (Chair, 2023-2024)

Data and Publication Committee (DPC)

Robert C. Green, MD, MPH	Harvard University (Chair)
Erin Drake	Harvard University

Resource Allocation Review Committee

Tom Montine, MD, PhD	University of Washington (Chair)
Cat Conti, BA	Northern California Institute for Research and Education

Administrative Core Leaders and Key Personnel

Michael W. Weiner, MD	University of California, San Francisco
Rachel Nosheny, PhD	University of California, San Francisco
Diana Truran Sacrey	Northern California Institute for Research and Education
Juliet Fockler	University of California, San Francisco
Melanie J. Miller, PhD	Northern California Institute for Research and Education
Catherine (Cat) Conti	Northern California Institute for Research and Education
Winnie Kwang, MA	University of California, San Francisco
Chengshi Jin, PhD	University of California, San Francisco
Adam Diaz, MS	Northern California Institute for Research and Education
Miriam Ashford, PhD	Northern California Institute for Research and Education
Derek Flenniken	Northern California Institute for Research and Education
Adrienne Kormos	Northern California Institute for Research and Education

Clinical Core Leaders and Key Personnel

Ronald Petersen, MD, PhD	Mayo Clinic, Rochester (Core PI)
Paul Aisen, MD	University of Southern California (Core PI)
Michael Rafii, MD, PhD	University of Southern California
Rema Raman, PhD	University of Southern California
Gustavo Jimenez, MBS	University of Southern California
Michael Donohue, PhD	University of Southern California
Jennifer Salazar, MBS	University of Southern California
Andrea Fidell, MPH	University of Southern California
Virginia Boatwright, BS	University of Southern California
Justin Robison, MS	University of Southern California
Caileigh Zimmerman, MS	University of Southern California
Yuliana Cabrera, BS	University of Southern California

Sarah Walter, MSc	University of Southern California
Taylor Clanton, MPH	University of Southern California
Elizabeth Shaffer, BS	University of Southern California
Caitlin Webb, BA	University of Southern California
Lindsey Hergesheimer, BS	University of Southern California
Stephanie Smith, BS	University of Southern California
Sheila Ogwang, MPH	University of Southern California
Olusegun Adegoke, MSc	University of Southern California
Payam Mahboubi, MPH	University of Southern California
Jeremy Pizzola, BA	University of Southern California
Cecily Jenkins, PhD	University of Southern California

Biostatistics Core Leaders and Key Personnel

Laurel Beckett, PhD	University of California, Davis (Core PI)
Danielle Harvey, PhD	University of California, Davis (Core PI)
Michael Donohue, PhD	University of Southern California
Naomi Saito, MS	University of California, Davis
Adam Diaz, MS	Northern California Institute for Research and Education
Kedir Adem Hussen, MS	University of Southern California

Engagement Core Leaders and Key Personnel

Ozioma Okonkwo, PhD	University of Wisconsin (Core-PI)
Monica Rivera-Mindt, PhD	Fordham University; Mt. Sinai (Core-PI)
Hannatu Amaza	University of Wisconsin
Mai Seng Thao	University of Wisconsin
Shaniya Parkins	Mt. Sinai
Omobolanle Ayo, MBChB, MPH	Mt. Sinai
Matt Glittenberg	University of Wisconsin
Isabella Hoang	University of Wisconsin
Kaori Kubo Germano, PhD	Fordham University
Joe Strong, PhD	University of Wisconsin
Trinity Weisensel	University of Wisconsin
Fabiola Magana	University of Wisconsin
Lisa Thomas	University of Wisconsin
Vanessa Guzman, PhD	Mt. Sinai
Adeyinka Ajayi, MBBS, MPH	Mt. Sinai
Joseph Di Benedetto, LMSW	Mt. Sinai
Sandra Talavera, MSW	Fordham University

MRI Core Leaders and Key Personnel

Clifford R. Jack, Jr., MD	Mayo Clinic, Rochester (Core PI)

Joel Felmlee, PhD	Mayo Clinic, Rochester
Nick C. Fox, MD	University College London
Paul Thompson, PhD	UCLA School of Medicine
Charles DeCarli, MD	University of California, Davis
Arvin Forghanian-Arani, PhD	Mayo Clinic, Rochester
Bret Borowski, RTR	Mayo Clinic, Rochester
Calvin Reyes	Mayo Clinic, Rochester
Caitie Hedberg	Mayo Clinic, Rochester
Chad Ward	Mayo Clinic, Rochester
Christopher Schwarz, PhD	Mayo Clinic, Rochester
Denise Reyes	Mayo Clinic, Rochester
Jeff Gunter, PhD	Mayo Clinic, Rochester
John Moore-Weiss, PhD	Mayo Clinic, Rochester
Kejal Kantarci, MD	Mayo Clinic, Rochester
Leonard Matoush	Mayo Clinic, Rochester
Matthew Senjem, MS	Mayo Clinic, Rochester
Prashanthi Vemuri, PhD	Mayo Clinic, Rochester
Robert Reid, PhD	Mayo Clinic, Rochester
Ian Malone, PhD	University College London
Sophia I. Thomopoulos, BS	University of Southern California School of Medicine
Talia M. Nir, PhD	University of Southern California School of Medicine
Neda Jahanshad, PhD	University of Southern California School of Medicine
Alexander Knaack, MS	University of California, Davis
Evan Fletcher, PhD	University of California, Davis
Danielle Harvey, PhD	University of California, Davis
Duygu Tosun-Turgut, PhD	University of California, San Francisco
Stephanie Rossi Chen, BA.	Northern California Institute for Research and Education
Mark Choe, BS	Northern California Institute for Research and Education
Karen Crawford	University of Southern California School of Medicine
Paul A. Yushkevich, PhD	University of Pennsylvania
Sandhitsu Das, PhD	University of Pennsylvania

PET Core Leaders and Key Personnel

William Jagust, MD	University of California, Berkeley (Core PI)
Susan Landau, PhD	University of California, Berkeley (Core PI)
Robert A. Koeppe, PhD	University of Michigan
Gil Rabinovici, MD	University of California San Francisco
Victor Villemagne, MD	University of Pittsburgh
Brian LoPresti, MSNE	University of Pittsburgh

Neuropathology Core Leaders and Key Personnel

Richard J. Perrin, MD, PhD	Washington University St. Louis (Core PI)
John Morris, MD	Washington University St. Louis
Erin Franklin, MS	Washington University St. Louis

Haley Bernhardt, BA, R. EEG T.	Washington University St. Louis
Nigel J. Cairns, PhD, MRCPath	Washington University St. Louis
Lisa Taylor-Reinwald, BA, HTL (ASCP)	Washington University St. Louis

Biomarkers Core Leader and Key Personnel

Leslie Shaw, PhD	UPenn School of Medicine (Core PI)
Edward B. Lee, MD, PhD	University of Pennsylvania (Core PI)
Virginia M.Y. Lee, PhD, MBA	UPenn School of Medicine
Magdalena Korecka, PhD	UPenn School of Medicine
Magdalena Brylska, MS	UPenn School of Medicine
Yang Wan, MS	UPenn School of Medicine
J.Q. Trojanowki, MD, PhD*	UPenn School of Medicine (*former Core PI, deceased)

Informatics Core Leader and Key Personnel

Arthur W. Toga, PhD	University of Southern California (Core PI)
Karen Crawford, MLIS	University of Southern California
Scott Neu, PhD	University of Southern California

Genetics Core Leader and Key Personnel

Andrew J. Saykin, PsyD	Indiana University School of Medicine (Core PI)
Kwangsik Nho, PhD	Indiana University School of Medicine (Core PI)
Tatiana M. Foroud, PhD	Indiana University School of Medicine (Dir. NCRAD)
Taeho Jo, PhD	Indiana University School of Medicine
Shannon L. Risacher, PhD	Indiana University School of Medicine
Hannah Craft, MPH	Indiana University School of Medicine
Liana G. Apostolova, MD	Indiana University School of Medicine
Kelly Nudelman, PhD	NCRAD/Indiana University School of Medicine
Kelley Faber, MS, CCRC	NCRAD/Indiana University School of Medicine
Zoë Potter, BA, CCRP	NCRAD/Indiana University School of Medicine
Kaci Lacy, MPH, CCRP	NCRAD/Indiana University School of Medicine
Rima Kaddurah-Daouk, PhD	Duke University/AD Metabolomics Consortium
Li Shen, PhD	University of Pennsylvania

ADNI4 Amyloid PET Visual Read Team

David Soleimani-Meigooni, MD	University of California, San Francisco
Renaud La Joie, PhD	University of California, San Francisco
Konstantinos Chiotis, MD, PhD	University of California, San Francisco
Maison Abu Raya, MD	University of California, San Francisco
Agathe Vrillon, MD, PhD	University of California, San Francisco
Charles Windon, MD	University of California, San Francisco
Julien Lagarde, MD, PhD	University of California, San Francisco
Zoe Lin	University of California, San Francisco
Aidyn Rose Hills	University of California, San Francisco

ADNI4 Amyloid Disclosure Team

Jason Karlawish, MD	University of Pennsylvania
Claire Erickson, PhD	University of Pennsylvania
Joshua Grill PhD	University of California, Irvine
Emily Largent PhD	University of Pennsylvania
Kristin Harkins MPH	University of Pennsylvania

Early Project Development

Michael W. Weiner, MD	UCSF/NCIRE
Leon Thal, MD – Past Investigator	
Zaven Kachaturian, PhD	Khachaturian, Radebaugh & Associates (KRA), Inc
Richard Frank, MD, PhD	General Electric
Peter J. Snyder, PhD	University of Connecticut

Alzheimer's Association's Ronald and Nancy Reagan's Research Institute

NIA

Neil Buckholtz, PhD	National Institute on Aging
John K. Hsiao, MD	National Institute on Aging
Laurie Ryan, PhD	National Institute on Aging
Susan Molchan, PhD	National Institute on Aging/National Institutes of Health

ADNI External Scientific Advisory Board (SAB)

Zaven Khachaturian, PhD	Prevent Alzheimer's Disease 2020 (Chair)
Maria Carrillo, PhD	Alzheimer's Association
William Potter, MD	National Institute of Mental Health
Lisa Barnes, PhD	Rush University
Marie Bernard, MD	NIA
Hector González	University of California, San Diego
Carole Ho	Denali Therapeutics
John K. Hsiao, MD	NIH
Jonathan Jackson, PhD	Massachusetts General Hospital
Eliezer Masliah, MD	NIA
Donna Masterman, MD	Biogen
Ozioma Okonkwo, PhD	University of Wisconsin, Madison
Richard Perrin, MD, PhD	Washington University St. Louis
Laurie Ryan, PhD	NIA
Nina Silverberg, PhD	NIA

Part B: Investigators By Site

Oregon Health and Science University:

Lisa Silbert, MD
Jeffrey Kaye, MD
Sylvia White (Salazar), ND
Aimee Pierce, MD
Amy Thomas, BSN, RN
Tera Clay
Daniel Schwartz, BA
Gillian Devereux, RN, MPH
Janet "Janae" Taylor
Jennifer Ryan, ND, MS
Mike Nguyen
Madison DeCapo, BS
Yanan Shang, MD

University of Southern California:
Lon Schneider, MD
Cynthia Munoz, MA
Diana Ferman, PA
Carlota Conant, BS
Katherin Martin
Kristin Oleary
Sonia Pawluczyk, MD
Elizabeth Trejo
Karen Dagerman
Liberty Teodoro, RN
Mauricio Becerra
Madiha Fairooz, BS
Sonia Garrison, MSsc
Julia Boudreau, MS
Yair Avila, BA

University of California--San Diego:
James Brewer, MD, PhD
Aaron Jacobson
Antonio Gama
Chi Kim
Emily Little, MPH
Jennifer Frascino
Nichol Ferng
Socorro Trujillo, MPH

University of Michigan:
Judith Heidebrink, MD
Robert Koeppe, PhD
Steven MacDonald, MD

Dariya Malyarenko, Ph.D.
Jaimie Ziolkowski, MA, BS, TLLP
James O'Connor, MS, RT (R)(MR)
Nicole Robert
Suzan Lowe
Virginia Rogers

Mayo Clinic, Rochester:
Ronald Petersen, MD, Ph.D.
Barbara Hackenmiller
Bradley Boeve, MD
Colleen Albers, RN
Connie Kreuger
David Jones, MD
David Knopman, MD
Hugo Botha, MB, Ch.B.
Jessica Magnuson
Jonathan Graff-Radford, MD
Kerry Crawley, BSW, CCRP
Michael Schumacher, CNMT
Sanna McKinzie, MS
Steven Smith, MS
Tascha Helland, BS
Val Lowe, MD
Vijay Ramanan, MD, PhD

Baylor College of Medicine:
Valory Pavlik, PhD
Jacob Faircloth, BS
Jeffrey Bishop, PA
Jessica Nath
Maria Chaudhary, MAP
Maria Kataki, PhD, MD
Melissa Yu, MD, FAAN
Nathiel Pacini, MA
Randall Barker
Regan Brooks, BA
Ruchi Aggarwal, MD

Columbia University Medical Center:
Lawrence Honig, MD, Ph.D.
Yaakov Stern, PhD

Akiva Mintz, MD
Jonathan Cordona, ARRT
Michelle Hernandez

Washington University, St. Louis:
Justin Long, MD
Abbey Arnold, NP
Alex Groves
Anna Middleton, RN
Blake Vogler
Cierra McCurry
Connie Mayo, RN
Cyrus Raji, MD, PhD
Fatima S. Amtashar, BS
Heather Klemp, MSW
Heather Nicole Elmore, RN, MSN, ANP-BC, CCRP
James Ruszkiewicz, CNMT
Jasmina Kusuran
Jasmine Stewart
Jennifer Horenkamp, RN, BSN
Julia Greeson, MS
Kara Wever, MA
Katie Vo, MD
Kelly Larkin, RN
Lesley Rao, MD
Lisa Schoolcraft, BFA
Lora Gallagher
Madeline Paczynski, BS, PA-C
Maureen McMillan
Michael Holt, MSW
Nicole Gagliano, BS, RT
Rachel Henson, MS
Renee LaBarge
Robert Swarm, MD
Sarah Munie, BSN, RN
Serena Cepeda, BS
Stacey Winterton, BSN, RN
Stephen Hegedus
TaNisha Wilson
Tanya Harte, FNP-BC
Zach Bonacorsi

University of Alabama Birmingham:
David Geldmacher, MD

Amber Watkins, RN
Brandi Barger, BSRT
Bryan Smelser, MD
Charna Bates, MA
Cynthia Stover, PENDING
Emily McKinley,
Gregory Ikner, MA
Haley Hendrix,
Harold Matthew Cooper, MSN, CRNP, NPC
Jennifer Mahaffey,
Lindsey Booth Robbins, MSN, CRNP, PNP-C
Loren Brown Ashley, RN, BSN
Marissa Natelson-Love, MD
Princess Carter, RN
Veronika Solomon,

Mount Sinai School of Medicine:
Hillel Grossman, MD
Alexandra Groome, BA
Allison Ardolino, MA
Anthony Kaplan, ARRT, CNMT
Faye Sheppard, BS
Genesis Burgos-Rivera, BA
Gina Garcia-Camilo, MD
Joanne Lim, MA
Judith Neugroschl, MD
Kimberly Jackson, BS
Kirsten Evans, BS
Laili Soleimani, MD
Mary Sano, Ph.D.
Nasrin Ghesani, MD
Sarah Binder, BS
Xiomara Mendoza Apuango, BS

Rush University Medical Center:
Ajay Sood, MD, PhD
Amelia Troutman, MA
Kimberly Blanchard, APRN, DNP, NP-C
Arlene Richards,
Grace Nelson, BA
Kirsten Hendrickson, RN, MSN
Erin Yurko,
Jamie Plenge, BS
Victoria Rufo, MS
Raj Shah, MD

Wein Center:
Ranjan Duara, MD
Brendan Lynch, CRT
Cesar Chirinos, PsyD
Christine Dittrich, CRT
Debbie Campbell
Diego Mejia, CRT
Gilberto Perez, CRT
Helena Colvee, BS
Joanna Gonzalez, PsyD
Josalen Gondrez, MS
Joshua Knaack
Mara Acevedo
Maria Cereijo, APRN
Maria Greig-Custo, MD
Michelle Villar, BS
Morris Wishnia
Sheryl Detling
Warren Barker, MS

Johns Hopkins University:
Marilyn Albert, Ph.D.
Abhay Moghekar
Barbara Rodzon
Corey Demsky
Gregory Pontone, MD
Jim Pekar
Leonie Farrington, CNRN
Martin Pomper
Nicole Johnson
Tolulope Alo

New York University:
Martin Sadowski, MD, PhD
Anaztasia Ulysse, BA
Arjun Masurkar
Brittany Marti
David Mossa, R.T
Emilie Geesey
Emily Petrocca, NP
Evan Schulze, PhD
Jennifer Wong
Joseph Boonsiri
Sunnie Kenowsky, DVM

Tatianne Martinez, NP
Veronica Briglall

Duke University Medical Center:
P. Murali Doraiswamy, MD, MBBS
Adaora Nwosu
Alisa Adhikari, BS
Cammie Hellegers, MA
Jeffrey Petrella
Olga James, MD
Terence Wong
Thomas Hawk

University of Pennsylvania:
Sanjeev Vaishnavi, MD, PhD
Hannah McCoubrey, BA
Ilya Nasrallah, MD, PhD
Rachel Rovere, BA
Jeffrey Maneval, MD
Elizabeth Robinson, MA
Francisco Rivera, MS
Jade Uffelman, BS
Martha Combs, BS, MS
Patricia O'Donnell
Sara Manning, MD

University of Kentucky:
Richard King, MD
Alayne Nieto, BSN, RN
Amanda Glueck, PhD
Anjana Mandal
Audrie Swain
Bethanie Gamble, PhD, RN
Beverly Meacham, RT(R) (MR)
Denece Forenback, RN
Dorothy Ross, CCRP
Elizabeth Cheatham
Ellen Hartman
Gary Cornell
Jordan Harp, PhD
Laura Ashe
Laura Goins
Linda Watts, RN
Morgan Yazell
Prabin Mandal

Regan Buckler, BSN, RN
Sylvia Vincent
Triana Rudd

University of Pittsburgh:
Oscar Lopez, MD
Ann Arlene Malia
Caitlin Chiado, CRNP
Cary Zik
James Ruszkiewicz, CNMT
Kathleen Savage
Linda Fenice
MaryAnn Oakley, MA
Paige C Tacey, M.Ed.
Sarah Berman, MD, PhD
Sarah Bowser, CRNP
Stephen Hegedus
Xanthia Saganis

University of Rochester Medical Center:
Anton Porsteinsson, MD
Abigail Mathewson, RN, BSN
Asa Widman, BA
Bridget Holvey, BS
Emily Clark, DO
Esmeralda Morales, MS
Iris Young, PA-C
James Ruszkiewicz, CNMT
Kevin Hopkins, BS, CNMT, LNMT
Kimberly Martin, RN, BSN
Nancy Kowalski, RN, MS
Rebecca Hunt, BS
Roberta Calzavara, PhD
Russell Kurvach, BS, CCRP
Stephen D'Ambrosio, PA-C, MPAS

University of California, Irvine:
Gaby Thai, MD
Beatriz Vides, RN, MSN
Brigit Lieb, ARRT/CRT
Catherine McAdams-Ortiz, MSN, RN, A/GNP
Cyndy Toso
Ivan Mares, BS
Kathryn Moorlach

Luter Liu
Maria Corona, PhD
Mary Nguyen, BA
Melanie Tallakson, DNP, FNP-C
Michelle McDonnell, PhD
Milagros Rangel, BS
Neetha Basheer, MD, MBBS
Patricia Place, BA
Romina Romero, PhD
Steven Tam, MD

University of Texas Southwestern Medical School:
Trung Nguyen, MD, PhD
Abey Thomas, ARRT
Alexander (Alex) Frolov, MD
Alka Khera, MD
Amy Browning, BA (Pending)
Brendan Kelley (031), MD
Courtney Dawson, RT(R)
Dana Mathews, MD, Ph.D.
Elaine Most, MS (Pending)
Elizeva (Ellie) Phillips, CNMT
Lynn Nguyen
Maribel Nunez
Matalin Miller, MS
Matthew R. Jones, MA
Natalie Martinez, MSN, RN, FNP-BC
Rebecca Logan, PA-C
Roderick McColl
Sari Pham
Tiffani Fox, MBA, MS
Tracey Moore, BA

Emory University:
Allan Levey, MD, PhD
Abby Brown, NP
Andrea Kippels, NP
Ashton Ellison, BSPH, ABA
Casie Lyons
Chadwick Hales, MD, PhD
Cindy Parry, BFA
Courtney Williams
Elizabeth McCorkle, BS
Guy Harris, BA

Heather Rose, BSN
Inara Jooma, BS
Jahmila Al-Amin, MS, BS
James Lah, MD, PhD
James Webster, BS
Jessica Swiniarski, MPH, BS
Latasha Chapman, BS
Laura Donnelly, MPH
Lauren Mariotti
Mary Locke, BS
Phyllis Vaughn, BSN
Rachael Penn, BSN, RN
Sallie Carpentier, RN, BSN
Samira Yeboah, BMSc, R.T.(R) (MR)
Sarah Basadre, BMSc, ARRT(R)(MR)
Sarah Malakauskas, MS
Stefka Lyron, NP
Tara Villinger, NP
Terra Burney

University of Kansas, Medical Center:
Jeffrey Burns, MD, MS
Ala Abusalim, PA-C
Alexandra Dahlgren, BS
Alexandria Montero, RN
Anne Arthur, BSN, MS, ANP-BC
Heather Dooly, BS
Katelynn Kreszyn, APRN
Katherine Berner, BS
Lindsey Gillen, APRN
Maria Scanlan, BA
Mercedes Madison, BS
Nicole Mathis
Phyllis Switzer
Ryan Townley, MD
Samantha Fikru, APRN, MSN, FNP-C
Samantha Sullivan, MSW
Ella Wright, BS

University of California, Los Angeles:
Maryam Beigi, MD
Anthony Daley
Ashley Ko
Brittney Luong
Glen Nyborg

Jessica Morales
Kelly Durbin, PhD
Lauren Garcia
Leila Parand
Lorena Macias
Lorena Monserratt, PhD
Maya Farchi
Pauline Wu, DO
Robert Hernandez
Thao Rodriguez, NP

Mayo Clinic, Jacksonville:
Neill Graff-Radford, MD, MBBCH, FRCP
A'llana Marolt, BS
Anton Thomas, BS
Deborah Aloszka
Ercilia Moncayo, BS
Erin Westerhold, RT
Gregory Day, MD
Kandise Chrestensen, BS
Mary Imhansiemhonehi, BS
Sanna McKinzie, MS
Sochenda Stephens, CCRP
Sylvia Grant, CCRC

Indiana University:
Jared Brosch, MD
Amy Perkins, CCRP
Aubree Saunders, BS
Debra Silberberg Kovac, BS
Heather Polson, CNMT
Isabell Mwaura, BS
Kassandra Mejia, BS
Katherine Britt, BS
Kathy King, RN
Kayla Nichols, BS
Kayley Lawrence, BA
Lisa Rankin, BSW
Martin Farlow, MD
Patricia Wiesenauer, MS
Robert Bryant, BS
Scott Herring, RN
Sheryl Lynch, RN
Skylar Wilson
Traci Day

William Korst

Yale University School of Medicine:
Christopher van Dyck, MD
Adam Mecca, MD, PhD
Alyssa Miller, BS
Amanda Brennan, LMSE, MSW
Amber Khan, MD
Audrey Ruan
Carol Gunnoud, AS
Chelsea Mendonca, MD
Danielle Raynes-Goldfinger, BS
Elaheh Salardini, MD
Elisa Hidalgo, MS, CNMT, EMT, RT (CT)
Emma Cooper, BA
Erawadi Singh, DO
Erin Murphy, BS
Jeanine May, APRN, MSN, MHP, CCRP
Jesse Stanhope, BS
Jessica Lam, BSE
Julia Waszak, BS
Kimberly Nelsen, BA
Kimberly Sacaza, BS
Mayer Joshua Hasbani, MD
Meghan Donahue, BA
Ming-Kai Chen, MD, PhD
Nicole Barcelos, MS, MA
Paul Eigenberger, MD
Robin Bonomi, MD
Ryan O'Dell, MD, PhD
Sarah Jefferson, MD
Siddharth Khasnavis, MD
Stephen Smilowitz, MD
Susan DeStefano, APRN, MSN
Susan Good, APRN
Terry Camarro, RT, RN, MRI, APRT
Vanessa Clayton, BS
Yanis Cavrel, BA
YuQuan "Oliver" Lu

McGill University, Montreal-Jewish General Hospital:
Howard Chertkow, MD
Howard Bergman, MD
Chris Hosein, M.Ed

Sunnybrook Health Sciences, Ontario:
Sandra Black, MD
Anish Kapadia, MD
Aparna Bhan
Benjamin Lam, MD, FRCP(c)
Christopher Scott, BSc
Gillian Gabriel, MA
Jennifer Bray, BA, BSW, MSW
Ljubica Zotovic, MD
Maria Samira Gutierrez
Mario Masellis
Marjan Farshadi, MD
Maurylette Gui, Psych BSc
Meghan Mitchell, BSc
Rebecca Taylor
Ruby Endre, M.R.T
Zhala Taghi-Zada

University of British Columbia Clinic for AD & Related Disorders
Robin Hsiung, MD
Carolyn English
Ellen Kim, BA
Eugene Yau
Haley Tong
Laura Barlow, RTR/RTMR
Lauren Jennings
Michele Assaly
Paula Nunes, PhD
Tahlee Marian

Cognitive Neurology St. Joseph's Ontario:
Andrew Kertesz, MD
John Rogers, MD
Dick Trost, PhD

Cleveland Clinic Lou Ruvo Center for Brain Health
Dylan Wint, MD
Charles Bernick, MD
Donna Munic, PhD

Northwestern University:
Ian Grant, MD
Aaliyah Korkoyah, BS
Ali Raja
Allison Lapins, MD

Caila Ryan, MS
Jelena Pejic
Kailey Basham, BS
Leena Lukose, BS
Loreece Haddad, MS
Lucas Quinlan, BS, MLS (ASCP)
Nathaniel Houghtaling

Premiere Research Inst (Palm Beach Neurology):
Carl Sadowsky MD
Walter Martinez MD
Teresa Villena MD

Georgetown University Medical Center:
Brigid Reynolds, NP
Angelica Forero, MS
Carolyn Ward, MSPH
Emma Brennan, BS
Esteban Figueroa
Giuseppe Esposito, MD
Jessica Mallory
Kathleen Johnson, RN, NP
Kathryn Turner, BSN
Katie Seidenberg
Kelly McCann, BA
Margaret Bassett, NP
Melanie Chadwick, NP
Raymond Scott Turner, MD, PhD
Robin Bean, RT
Saurabh Sharma, MD

Brigham and Women's Hospital:
Gad Marshall, MD
Aferdita Haviari, BA
Alison Pietras, PA-C, ACP
Bradley Wallace, BS
Catherine Munro, PhD
Gladiliz Rivera-Delpin, MA
Hadley Hustead, BS
Isabella Levesque
Jennifer Ramirez, BA
Karen Nolan, BS, RT (MR)
Kirsten Glennon, RN, CNRN
Mariana Palou, BA
Michael Erkkinen, MD

Nicole DaSilva
Pamela Friedman, Psy. D
Regina M. Silver, RN
Ricardo Salazar, MD
Roxxanne Polleys, AA
Scott McGinnis (094), MD
Seth Gale, MD
Tia Hall, BS
Tuan Luu

Stanford University:
Steven Chao, MD
Emmeline Lin, BS
Jaila Coleman, BA
Kevin Epperson, RT(R)(MR)
Minal Vasanawala

Banner Sun Health Research Institute
Alireza Atri, MD, PhD
Amy Rangel
Brittani Evans
Candy Monarrez
Carol Cline, LMSW
Carolyn Liebsack, RN, BSN, CCRC
Daniel Bandy
Danielle Goldfarb, MD
Debbie Intorcia
Jennifer Olgin
Kelly Clark
Kelsey King, CCRP
Kylee York
Marina Reade, RN, FNP-C
Michael Callan
Michael Glass
Michaela Johnson, G-ACNP, BC
Michele Gutierrez
Molly Goddard
Nadira Trncic, MD, PhD
Parichita Choudhury, MD
Priscilla Reyes
Serena Lowery
Shaundra Hall
Sonia Olgin
Stephanie de Santiago, RN, NP

Boston University:
Michael Alosco, PhD
Alyssa Ton, BS
Amanda Jimenez, MS, EMT-B, CPT
Andrew Ellison, MR Technologist
Anh Tran, RN
Brandon Anderson, RT(N), CNMT
Della Carter, MS
Donna Veronelli, RTN, CNMT
Steven Lenio, MD
Eric Steinberg, RN, MSN, CNP
Jesse Mez, MD, MS
Jason Weller, MD
Jennifer Johns, RN
Jesse Mez, MD, MS
Jessica Harkins, CNMT
Alexa Puleio, MS
Ina Hoti, BS
Jane Mwicigi, MBChB., MPH
Alexa Puleio, MS
Michael Alosco, PhD
Olivia Schultz, BA
Mona Lauture, RN
Eric Steinberg
Ridiane Denis, RN
Ronald Killiany, PhD
Sarab Singh, CNMT
Steven Lenio, MD
Wendy Qiu, MD, PhD
Ycar Devis, MPH

Howard University:
Thomas Obisesan, MD, MPH
Andrew Stone, MS
Debra Ordor, RN, BSN
Ifreke Udodong, CRNP
Immaculata Okonkwo, DNP, MSN, APRN, FNP-BC
Javed Khan, MD
Jillian Turner, BS, MS
Kyliah Hughes, BS, RMA
Oshoze Kadiri, MPH

Case Western Reserve University:
Charles Duffy, MD, PhD

Ariana Moss
Katherine Stapleton, LPN
Maria Toth (fmr Gross), RN
Marianne Sanders, BSN, RN
Martin Ayres
Melissa Hamski
Parianne Fatica, CCRC
Paula Ogrocki, PhD
Sarah Ash
Stacy Pot

University of California, Davis Sacramento :
Doris Chen, MD
Andres Soto
Costin Tanase, PhD
David Bissig, MD, PhD
Hafsanoor Vanya, BA
Heather Russell (116), CNMT
Hitesh Patel, CNMT
Hongzheng Zhang, CCRP
Kelly Wallace, CCRP
Kristi Ayers, BS
Maria Gallegos, BS
Martha Forloines, PhD
Meghan Sinn
Queennie Majorie S Kahulugan, CCRC
Richard Isip, RT (R)(N)(CT)
Sandra Calderon, MS, RN, FMP-C
Talia Hamm, BA, CCRP

Parkwood Hospital:
Michael Borrie, MD
T-Y Lee, PhD
Dr Rob Bartha, PhD

University of Wisconsin:
Sterling Johnson, PhD
Sanjay Asthana, MD
Cynthia M. Carlsson, MD

Banner Alzheimer's Institute:
Allison Perrin, MD
Pierre Tariot, MD
Adam Fleisher, MD
Stephanie Reeder, BA

Dent Neurologic Institute
Horacio Capote, MD
Allison Emborsky
Anna Mattle, PharmD, MS
Bela Ajtai, MD
Benjamin Wagner, PA-C
Bennett Myers
Daryn Slazyk
Delaney Fragale, PA-C
Erin Fransen, PA
Heather Macnamara
Jonathan Falletta, PA-C
Joseph Hirtreiter, RN
Laszlo Mechtler, MD
Megan King
Michael Asbach, RPA-C
Michelle Rainka, Pharm. D., CCRP
Richard Zawislak, NP
Scott Wisniewski
Stephanie O'Malley, PA-C
Tatiana Jimenez-Knight
Todd Peehler
Traci Aladeen, PharmD
Vernice Bates
Violet Wenner
Wisam Elmalik, MD

Ohio State University:
Douglas W. Scharre, MD
Arun Ramamurthy, MD
Soumya Bouchachi, MD
Maria Kataki, MD, PhD - Past Investigator
Rawan Tarawneh, MD - Past Investigator
Brendan Kelley, MD - Past Investigator

Albany Medical College:
Dzintra Celmins, MD
Alicia Leader
Chris Figueroa
Heather Bauerle, NP
Katlynn Patterson
Michael Reposa
Steven Presto
Tuba Ahmed

Wendy Stewart

Hartford Hosp, Olin Neuropsychiatry Research Center:
Godfrey D. Pearlson MD
Karen Blank, MD
Karen Anderson, RN

Dartmouth-Hitchcock Medical Center:
Robert B. Santulli, MD
Eben S. Schwartz, PhD

Wake Forest University Health Sciences:
Jeff Williamson, MD, MHS, FACP
Alicia Jessup, RN
Andrea Williams
Crystal Duncan
Abigail O'Connell, APRN, FNP-C
Karen Gagnon
Ezequiel Zamora
James Bateman
Freda Crawford, CNMT
Deb Thompson
Eboni Walker
Jennifer Rowell
Mikell White, MHA
Phillip "Hunter" Ledford
Sarah Bohlman, MSL
Susan Henkle, RN
Joseph Bottoms, CNMT
Lena Moretz, RT(R) CT (MR)
Bevan Hoover, BS
Michael Shannon
Samantha Rogers, PA-C
Wendy Baker
William Harrison, MD

Rhode Island Hospital:
Chuang-Kuo Wu, MD
Alexis DeMarco, BS
Ava Stipanovich, BS, ScM
Daniel Arcuri, CNMT, RT(N)(CT)
Jan Clark, RN, BSN, CCRC, CSNT
Jennifer Davis, PhD
Kerstin Doyon, RN, BSN

Marie Amoyaw, BA
Mauro Veras Acosta, PENDING, BS
Ronald Bailey, RT-R, CNMT
Scott Warren, MD
Terry Fogerty
Victoria Sanborn, PhD

Butler Hospital
Meghan Riddle, MD
Stephen Salloway, MD, MS
Paul Malloy, PhD
Stephen Correia, PhD

University of California San Francisco
Charles Windon, MD
Morgan Blackburn
Howard J. Rosen, MD
Bruce L. Miller, MD

University of South Florida, Byrd Institute
Amanda Smith, MD
Ijeoma Mba, MBA, MPH
Jenny Echevarria
Juris Janavs

University of Chicago
Emily Roglaski, PhD
Meagan Yong
Rebecca Devine

Eastern Virginia Medical School
Hamid Okhravi, MD

Charter Health Research Services
Edgardo Rivera, MD
Teresa Kalowsky
Caroline Smith
Christina Rosario

Houston Methodist Neurological Institute
Joseph Masdeu, MD, PhD
Richard Le, PharmD
Maushami Gurung

Barrow Neurological Institute
Marwan Sabbagh, MD
Angelica Garcia
Micah Ellis Slaughter
Nadeen Elayan
Skieff Acothley

Nathan Kline Institute
Nunzio Pomara, MD
Raymundo Hernando
Vita Pomara
Chelsea Reichert

Ralph Johnson Veterans Administration Health Care Services
Olga Brawman-Mintzer, MD
Allison Acree
Arthur Williams
Campbell Long
Rebecca Long

Vanderbilt University Medical Center
Paul Newhouse, MD
Sydni Jenee Hill
Amy Boegel

University of Texas Health, San Antonio
Sudha Seshadri, MD
Amy Saklad
Floyd Jones

Rutgers University
William Hu, MD, PhD
V. Sotelo

Gonzalez & Aswad Health Services
Yaneicy Gonazalez Rojas, MD

Medical University South Carolina
Jacobo Mintzer, MD, MBA
Crystal Flynn Longmire, PhD
Kenneth Spicer, MD, PhD
