# Supplementary material for: Hemispheric asymmetry of tau pathology is related to asymmetric amyloid deposition in Alzheimer’s Disease
Source: Nat Commun. 2025 Sep 5;16:8232. doi: 10.1038/s41467-025-63564-2 (PMC12413461; doi:10.1038/s41467-025-63564-2)
Supplement: Supplementary file 2 — Reporting Summary [file 41467_2025_63564_MOESM2_ESM.pdf]

nature portfolio

Corresponding author(s): Toomas Erik Anijärv

Last updated by author(s): Aug 5, 2025

Reporting Summary

Nature Portfolio wishes to improve the reproducibility of the work that we publish. This form provides structure for consistency and transparency in reporting. For further information on Nature Portfolio policies, see our Editorial Policies and the Editorial Policy Checklist.

Statistics

For all statistical analyses, confirm that the following items are present in the figure legend, table legend, main text, or Methods section.

n/a	Confirmed	
☐	☒	The exact sample size (*n*) for each experimental group/condition, given as a discrete number and unit of measurement
☐	☒	A statement on whether measurements were taken from distinct samples or whether the same sample was measured repeatedly
☐	☒	The statistical test(s) used AND whether they are one- or two-sided<br>*Only common tests should be described solely by name; describe more complex techniques in the Methods section.*
☐	☒	A description of all covariates tested
☐	☒	A description of any assumptions or corrections, such as tests of normality and adjustment for multiple comparisons
☐	☒	A full description of the statistical parameters including central tendency (e.g. means) or other basic estimates (e.g. regression coefficient) AND variation (e.g. standard deviation) or associated estimates of uncertainty (e.g. confidence intervals)
☐	☒	For null hypothesis testing, the test statistic (e.g. F, t, r) with confidence intervals, effect sizes, degrees of freedom and P value noted<br>*Give P values as exact values whenever suitable.*
☒	☐	For Bayesian analysis, information on the choice of priors and Markov chain Monte Carlo settings
☐	☒	For hierarchical and complex designs, identification of the appropriate level for tests and full reporting of outcomes
☐	☒	Estimates of effect sizes (e.g. Cohen's *d*, Pearson's *r*), indicating how they were calculated

Our web collection on statistics for biologists contains articles on many of the points above.

Software and code

Policy information about availability of computer code

Data collection	Computer code regarding data collection is performed not directly available within this manuscript, but more detailed description of the main cohort used in this study can be found in https://biofinder.se/ (NCT03174938)
Data analysis	Data analyses scripts (in Python-based Jupyter Notebooks) are publicly available on GitHub (https://github.com/teanijarv/phd-neurodegeneration-imaging/tree/main/projects/01_tau_asymmetry). All notebooks show each step of analysis with the figures used in the paper generated in the notebook.

For manuscripts utilizing custom algorithms or software that are central to the research but not yet described in published literature, software must be made available to editors and reviewers. We strongly encourage code deposition in a community repository (e.g. GitHub). See the Nature Portfolio guidelines for submitting code & software for further information.

Data

Policy information about availability of data

All manuscripts must include a data availability statement. This statement should provide the following information, where applicable:

- Accession codes, unique identifiers, or web links for publicly available datasets
- A description of any restrictions on data availability
- For clinical datasets or third party data, please ensure that the statement adheres to our policy

Four different cohorts were used in this study: BioFINDER-2, ADNI, A4, and OASIS-3. For BioFINDER-2 data, anonymized data will be shared by request from a

qualified academic investigator as long as data transfer is in agreement with European Union legislation on the general data protection regulation and decisions by the Swedish Ethical Review Authority and Region Skåne, which should be regulated in a material transfer agreement. ADNI, A4 and OASIS3 are publicly available datasets and can be obtained from http://adni.loni.usc.edu/, https://ida.loni.usc.edu/ and https://sites.wustl.edu/oasisbrains/, respectively.

Research involving human participants, their data, or biological material

Policy information about studies with human participants or human data. See also policy information about sex, gender (identity/presentation), and sexual orientation and race, ethnicity and racism.

Reporting on sex and gender	The BioFinder-2 cohort (the main study population) does not include any non-binary participants. Nevertheless, this study used only the biological sex as a variable. All the external cohorts used for replication purposes to our knowledge report biological sex.
Reporting on race, ethnicity, or other socially relevant groupings	Race, ethnicity, or other socially relevant groupings were not included in this study as covariates due to the main study population being mainly white Swedish people.
Population characteristics	The main study population included elderly participants around 70-80 years of age who were displaying Alzheimer's disease (AD) related pathological progression and were diagnosed as either cognitively unimpaired, mildly cognitively impaired, or having AD dementia - patients with other neurodegenerative diseases diagnosed as main diagnosis were excluded. Moreover, patients with serious other neurological conditions (such as brain tumor) were also not included in the study sample. By sex, the study population was relatively balanced with numerically small bias to having more females than males. Furthermore, education years and APOE2/3/4 carriership was measured.
Recruitment	Recruitment of the participants was done by separate clinical team, who were not part of the analysis process. All particpants were recruited during the process clinical procedures at a memory clinic in Malmö, Sweden and given an option to participate in the BioFINDER-2 cohort and asked to give consent to be involved in future studies using the data of this cohort. In detail, the Swedish BioFINDER-2 study enrolls participants in five sub-cohorts. Cohort A and B includes neurologically and cognitively healthy controls. The inclusion criteria are: i) ages 40-65 years (cohort A) and ages 66-100 years (cohort B); ii) absence of cognitive symptoms as assessed by a physician with special interest in cognitive disorders; iii) Mini Mental State Examination (MMSE) score 27-30 (cohort A) or 26-30 (cohort B) at screening visit; iv) do not fulfill the criteria for MCI or any dementia according to DSM-51; v) fluent in Swedish. The participants in the present study had been enrolled in either cohort A, B, or C (only people with SCD) of the BioFINDER-2 study for the cognitively unimpaired (CU) group, in cohort C for the MCI group, or D for the AD group. All participants were assessed by physicians with expertise in dementia disorders.
Ethics oversight	All participants gave written informed consent to participate in the BioFINDER-2 study as approved by the ethics committee of Lund University, Sweden.

Note that full information on the approval of the study protocol must also be provided in the manuscript.

Field-specific reporting

Please select the one below that is the best fit for your research. If you are not sure, read the appropriate sections before making your selection.

☒ Life sciences ☐ Behavioural & social sciences ☐ Ecological, evolutionary & environmental sciences

For a reference copy of the document with all sections, see nature.com/documents/nr-reporting-summary-flat.pdf

Life sciences study design

All studies must disclose on these points even when the disclosure is negative.

Sample size	A total of 837 participants were included in this study which was based on the maximum availability of participants from the BioFINDER-2 study cohort.
Data exclusions	Besides the participants inclusion/exclusion criteria which mentioned briefly before, during the analysis a few subjects were excluded due to quality control check at analyses done with MRI data (e.g., during white matter tract segmentations, some DWI scans were of poor quality), therefore they were not included in the corresponding analyses.
Replication	The main findings of this study were replicated in three external cohorts - Open Access Series of Imaging Studies (OASIS-3) Anti-Amyloid Treatment in Asymptomatic Alzheimer's Disease (A4), and Alzheimer's Disease Neuroimaging Initiative (ADNI).
Randomization	N/A
Blinding	Clinical personnel who collected the data were not part of the study analysis.

Reporting for specific materials, systems and methods

We require information from authors about some types of materials, experimental systems and methods used in many studies. Here, indicate whether each material, system or method listed is relevant to your study. If you are not sure if a list item applies to your research, read the appropriate section before selecting a response.

Materials & experimental systems

n/a	Involved in the study
☒	☐ Antibodies
☒	☐ Eukaryotic cell lines
☒	☐ Palaeontology and archaeology
☒	☐ Animals and other organisms
☐	☒ Clinical data
☒	☐ Dual use research of concern
☒	☐ Plants

Methods

n/a	Involved in the study
☒	☐ ChIP-seq
☒	☐ Flow cytometry
☐	☒ MRI-based neuroimaging

Clinical data

Policy information about clinical studies

All manuscripts should comply with the ICMJE guidelines for publication of clinical research and a completed CONSORT checklist must be included with all submissions.

Clinical trial registration: NCT03174938

Study protocol: https://www.clinicaltrials.gov/study/NCT03174938?term=BioFINDER&rank=1 / https://biofinder.se/two/

Data collection: BioFINDER-2 data collection was performed in a memory clinic in Malmö, Sweden.

Outcomes: The study-specific aims were descriptive: to find associations between hemispheric asymmetry in tau pathology distribution and other spatial characteristics of the brain.
However in general, the BioFINDER-2 study has five main aims which all are not relevant to this specific work:
- Develop methods for improved diagnostic and prognostic work-up of different dementia disorders
- Develop biomarkers and imaging techniques to monitor early effects of new disease-modifying therapies
- Investigate the heterogeneity of dementia and parkinsonian disorders to assist in the development of a new pathology-based disease classification
- Define the temporal evolution of pathologies in the predementia phases of Alzheimer's disease
- Investigate the underlying disease mechanisms of dementia disorders in humans aiming at finding new relevant drug targets

Plants

Seed stocks: *Report on the source of all seed stocks or other plant material used. If applicable, state the seed stock centre and catalogue number. If plant specimens were collected from the field, describe the collection location, date and sampling procedures.*

Novel plant genotypes: *Describe the methods by which all novel plant genotypes were produced. This includes those generated by transgenic approaches, gene editing, chemical/radiation-based mutagenesis and hybridization. For transgenic lines, describe the transformation method, the number of independent lines analyzed and the generation upon which experiments were performed. For gene-edited lines, describe the editor used, the endogenous sequence targeted for editing, the targeting guide RNA sequence (if applicable) and how the editor was applied.*

Authentication: *Describe any authentication procedures for each seed stock used or novel genotype generated. Describe any experiments used to assess the effect of a mutation and, where applicable, how potential secondary effects (e.g. second site T-DNA insertions, mosiacism, off-target gene editing) were examined.*

Magnetic resonance imaging

Experimental design

Design type: Resting-state

Design specifications: A 3 Tesla MRI (Siemens Prisma) is conducted in the BioFINDER-2 cohort. We employ a variety of MRI techniques to assess regional brain volume (3D MPRAGE), metabolism (MRS), structural and functional connectivity (dMRI and fMRI), regional blood flow (ASL), iron deposition (SWI), and the presence of small vessel disease (using MPRAGE, SWI, and FLAIR). This comprehensive protocol takes approximately 60 minutes to complete, and no contrast agent is used.
In this manuscript, 3D MPRAGE, dMRI, resting-state fMRI, ASL modalities were used.

Behavioral performance measures: N/A

Acquisition

Imaging type(s): MRI, RSfMRI, dMRI, ASL

Field strength: 3T

Sequence & imaging parameters: The MRI imaging was conducted using a MAGNETOM Prisma 3T MRI scanner (Siemens Healthineers) with a 64-channel head coil. RSfMRI was acquired using a gradient-echo planar sequence (eyes closed; in-plane resolution=3×3mm2; slice thickness=3.6mm; repetition time=1020ms; echo time=30ms; flip-angle=63°; 462 dynamic scans over a period of 7.85min). For dMRI, 104 diffusion-weighted imaging volumes were acquired using a single-shot echo-planar imaging sequence (repetition time = 3500ms; echo time = 73ms; resolution = 2×2×2mm3; field of view = 220×220×124mm3; b-values range = 0, 100, 1000 and 2500s/mm2 distributed over 2, 6, 32 and 64 directions; 2-fold parallel acceleration and partial Fourier factor = 7/8). T1-weighted structural images were also acquired using a magnetization-prepared rapid gradient-echo (MPRAGE) sequence (inversion time = 1100ms; flip-angle = 9°; echo time = 2.54ms; echo spacing = 7.3ms; repetition time = 1900ms; receiver bandwidth = 220 Hz/pixel; voxel size = 1×1×1mm3). Generalized autocalibrating partially parallel acquisitions (GRAPPA) was applied with an acceleration factor of 2 and 24 reference lines. Additionally, ASL scans were acquired on a subset of the sample using a prototype 3D pseudo-continuous (pCASL) sequence with background suppression and gradient- and spin-echo (GRASE) readout.

Area of acquisition: Whole brain

Diffusion MRI: ☒ Used ☐ Not used

Parameters: 104 diffusion-weighted imaging volumes were acquired using a single-shot echo-planar imaging sequence (repetition time = 3500ms; echo time = 73ms; resolution = 2×2×2mm3; field of view = 220×220×124mm3; b-values range = 0, 100, 1000 and 2500s/mm2 distributed over 2, 6, 32 and 64 directions; 2-fold parallel acceleration and partial Fourier factor = 7/8).

Preprocessing

Preprocessing software:
FreeSurfer (version 6.0, https://surfer.nmr.mgh.harvard.edu)
FSL (FMRIB Software Library, version 6.0.4; Oxford, UK)
MRtrix3 (https://www.mrtrix.org/)
TractSeg (https://github.com/MIC-DKFZ/TractSeg)
Configurable Pipeline for the Analysis of Connectomes (C-PAC; https://fcp-indi.github.io/)
Network Based Statistic Toolbox (https://sites.google.com/site/bctnet/network-based-statistic-toolbox)

Normalization:
T1-weighted images were pre-processed with steps such as correction for intensity homogeneity, skull stripping, and tissue segmentation.
dMRI images underwent correction for susceptibility-induced distortions, using images acquired with opposite phase polarities, motion, and Eddy current induced artifacts.
RSfMRI data underwent preprocessing including motion correction, bandpass filtering (0.01-0.1 Hz), and noise regression. Susceptibility distortion was corrected using T2-based unwarping, and outlier frames identified by DVARS (D refers to temporal derivative of the time courses and VARS refers to RMS variance over voxels) were censored.

Normalization template: Subject space

Noise and artifact removal: Mentioned above

Volume censoring: Mentioned above

Statistical modeling & inference

Model type and settings:
Functional connectivity based on Pearson's correlation between regions using RSfMRI
Structural connectivity based on anatomically constrained tractography and diffusion tensor imaging using dMRI

Effect(s) tested: Connectivity between regions defined using Desikan-Killiany atlas (Freesurfer's aparcaseg)

Specify type of analysis: ☐ Whole brain ☐ ROI-based ☒ Both

Anatomical location(s):
Functional and structural connectivity analyses using whole-brain connectomes used all DK region-to-region connections that were inter-hemispheric and were averaged together. Furthermore, whole-brain connectome analysis was performed using Network Based Statistic method.
Microstructural integrity analysis used inter-hemispheric white matter tracts (corpus callosum, forceps major, forceps minor)

Statistic type for inference (See Eklund et al. 2016):
For the analyses using average inter-hemispheric functional or structural connectivity or tract-based structural connectivity, linear regressions were used to compare the values between groups.
Whole-brain connectome analysis using Network Based Statistic used a cluster-wise inference.

Correction: All analyses composing of multiple regions were corrected using FDR/FWE multiple comparisons correction

Models & analysis

n/a	Involved in the study
☐	☒ Functional and/or effective connectivity
☐	☒ Graph analysis
☐	☒ Multivariate modeling or predictive analysis

Functional and/or effective connectivity	Pearson's correlation
Graph analysis	Network Based Statistics
Multivariate modeling and predictive analysis	Anatomically constrained tractography
